# Metformin suppresses inflammation and apoptosis of myocardiocytes by inhibiting autophagy in a model of ischemia-reperfusion injury

**DOI:** 10.7150/ijbs.40823

**Published:** 2020-07-19

**Authors:** Kai-yu Huang, Jia-qun Que, Ze-song Hu, Yong-wei Yu, Ying-ying Zhou, Lei Wang, Yang-jing Xue, Kang-ting Ji, Xin-min Zhang

**Affiliations:** 1 Department of Cardiology, The Second Affiliated Hospital and Yuying Children's Hospital of Wenzhou Medical University, Wenzhou 325027, Zhejiang, China.; 2 The Second School of Medicine, Wenzhou Medical University, Wenzhou 325027, Zhejiang, China.; 3 Department of Endocrinology, The Second Affiliated Hospital and Yuying Children's Hospital of Wenzhou Medical University, Wenzhou 325027, Zhejiang, China.

**Keywords:** Metformin, autophagy, apoptosis, inflammation, myocardial ischemia reperfusion

## Abstract

Metformin (Met) is a major widely used oral glucose lowering drug for the treatment of type 2 diabetes. It is reported that metformin could regulate autophagy in various diseases of cardiovascular system including in I/R injury, diabetic cardiomyopathy and heart failure. Autophagy plays a controversial role in ischemia/reperfusion (I/R) injury, and this research was performed to explore the cardioprotective effect of Met on I/R injury and discuss the underlying mechanism of autophagy in it. *In vivo* and *in vitro*, Met exerted cardioprotection function of decreasing myocardial inflammation and apoptosis with a decrease in the level of autophagy. Moreover, Met significantly inhibited autophagosome formation and restore the impairment of autophagosome processing, which lead to cardioprotection effect of Met. Akt was up-regulated in Met-treated I/R hearts and miransertib, a pan-AKT inhibitor, was able to reverse the alleviating autophagy effect of Met. We demonstrate that Met protects cardiomyocytes from I/R-induced apoptosis and inflammation through down regulation of autophagy mediated by Akt signaling pathway.

## Introduction

Acute myocardial infarction (AMI) is one of the major diseases threatening human life in the world 1. The most effective treatment for myocardial infarction is coronary reperfusion therapy 2. Inevitably, the restoration of blood flow to the ischemic myocardium will result in myocardial damage, which we called myocardium I/R injury 3. Therefore, it is urgent to explore an effective therapeutic modality to prevent heart from I/R injury. Autophagy is a common physiological process in the heart. It mediates the normal turnover of damaged and dysfunctional organelles and protein 4 .Whether autophagy plays a beneficial or detrimental role during myocardial I/R remains a subject of controversy 4. Induction of autophagy correlated with cardiomyocyte death of the mouse heart during reperfusion 5, although some researches demonstrated that autophagy is protective to myocardium I/R 6. Met is one of the most widely used antihyperglycemic drugs for the treatment of type 2 diabetes 7. Many studies showed that Met improved cardiac function and reduces the myocardial injury in diabetic patients as well as mice suffered from ischemic cardiomyopathy 8, 9. The cardioprotective mechanism of Met is not limited to its hypoglycemic effect alone 10, and recent original researches showed many potential mechanisms including anti-apoptosis, inhibition of mitochondrial permeability transition pore (mPTP) opening, up-regulation of antioxidant enzymes 9, 11, 12. Numerous studies have clarified that Met was able to regulate the level of autophagy in many organs and diseases 13-15, but not yet in nondiabetic mice in a model of I/R injury, especially in a low-dose, single Met treatment. AMPK, which is widely believed to be activated by Met seems to have no distinct effect on the regulation of autophagy during reperfusion 16, 17. On the contrary, Akt is able to activate mTOR during reperfusion and then inhibit autophagy 16. We speculated that Met may have protective effects on myocardium I/R injury via autophagy regulation in an Akt-dependent manner.

Therefore, the aim of this study was to investigate the role of autophagy in the protection of Met against non-diabetic myocardial I/R injury, using an *in vivo* model of I/R and an *in vitro* model of H2O2-induced I/R injury. Our findings led to a new view that Met could compromise autophagy, thus dampen apoptosis, inflammation in non-diabetic myocardium and protected heart from I/R injury through activating Akt. This study provided us an insight into the mechanism of Met in protecting myocardial I/R injury under a new perspective.

## Materials and Methods

### Animals and Cell preparation

All experiments performed on animals were approved by Animal Care and Use Committee of Wenzhou Medical University and were in accordance with the ARRIVE guidelines for reporting experiments involving animals. Specific pathogen free (SPF) male C57BL/6 mice (20-25g), 7-8 weeks old, were obtained from the SLAC Laboratory Animal Centre of Shanghai. In each cage, 3-4 mice could take food and water at will under a 12 h light-dark cycles with a constant room temperature (25±2 °C) for one week before the experiment. All efforts were made to minimize the suffering of the animals and the number of animals. The results were based on the rule of the replacement, refinement or reduction (the 3Rs). H9C2 cells was obtained from the American Type Culture Collection (Manassas, VA, USA), were cultured in Dulbecco's Modified Eagle's Medium (DMEM) containing 4.5 g/L glucose, 10% fetal bovine serum (FBS), and 1% penicillin/streptomycin at 37°C in a humidified atmosphere. All of the above cells were passaged for less than 6 months from the day of recovery. Primary cultures of neonatal mouse cardiac myocytes were prepared as described previously 18.

### Myocardial I/R protocol

Surgical ligation of the left coronary artery (LCA) was performed as described previously 19. In short, we anesthetized mice with inhalation of isoflurane. Then the heart was cut laterally along the upper edge of the third or fourth rib. When the entire left anterior descending coronary artery (LAD) was exposed, we ligated the distal 1/3 of the LAD with a 7-0 silk suture. After 30 min of ischemia, the slipknot was released for 4 h (for analysis of protein expression, myocardial apoptosis and infarct size, the level of CK-MB in serum), 24h (for detection of myocardial pathological structural changes and inflammatory cell infiltrates) and 7 days (for myocardial fibrosis determination). Mice (n=6/group) were treated as follows. In the Met groups, 125µg/kg Met (dissolved in 100µL of saline) was intravenous administered 15 min before surgery 20. Rapamycin (an autophagosome formation activator, 0.25 mg/kg), chloroquine (CQ, a lysosomal acidification inhibitor, 10 mg/kg), Miransertib (a pan-AKT inhibitor, 120 mg/kg) or 3-MA (an autophagosome formation inhibitor, 30 mg/kg) was administered via an intraperitoneal injection 1 h before surgery. Sham group mice experienced the same protocol without LAD occlusion. After the surgery, the mice were immediately received 10 mg/ml of pentobarbital sodium (0.1 ml/20 g) to ensure minimal pain and moved to a quiet room under gentle lighting. At the end of reperfusion, mice were euthanized after isoflurane anesthesia.

### Cell treatment

When cells reached 70-80% confluence, subsequent experiments were performed. Briefly, H9C2 cells were cultured with 250µM H2O2 in DMEM containing 2% FBS for 4 h to induce I/R procedure. To evaluate the protective effects of Met and the role of autophagy, cells were pretreated with Met (50 μM), CQ (10 μM) for 12h, rapamycin (100 nM) and 3-MA (10 mM) for 2h. The exact group size for each experimental group *in vitro* is 6.

### CRISPR-Cas9-repressor assay

Akt knockout cultured primary cardiomyocytes were generated with CRISPR-Cas9 plasmid and crRNA (sequence: 5'-ACAGAGAAATTGTTCAGGGG-3') targeting host gene (Akt) expression vector. The CRISPR-Cas9 system was produced by Ribo-Bio (Ribo-Bio, Guangzhou, China). Experimental samples were obtained 72 h after transfection according to the directions of the manufacturer.

### Cell viability assay

Cell viability was assayed with the CCK-8 according to the manufacturer's protocol. In brief, H9C2 cells were planted in 96-well plates (5000 cells/ well) and incubated in DMEM with 10% FBS at 37 °C until confluence reached 70-80%. At first, various doses of H_2_O_2_ (50, 150, 250, 350, 450, 550 and 650 μM) were added to cardiomyocytes and incubated for 4 h to determine an appropriate condition of I/R procedure. On the other hand, Met, rapamycin, CQ and 3-MA were pretreated as described above. After treatment, the cells were washed with phosphate-buffered saline (PBS), and then cells were incubated in 10% CCK-8 solution (v/v) for an additional 2 h and cell viability was evaluated by CCK-8 assay. The amount of cell viability was normalized to the control group, which was considered as 100%.

### Staining of autophagic vacuoles by monodansylcadaverine (MDC)

Fluorescent probe MDC is a marker for autophagolysosomes to evaluate autophagy 21. Although MDC does not specifically label autophagosomes, it can quantify autophagy in conjunction with other autophagy markers 22. After various treatments, cells were exposed to 0.05 mM MDC for 30 min at 37 °C in the dark. Then, cells were washed with PBS for 3 times and observed immediately by fluorescence microscope (Olympus). In order to determine the relative level of MDC, values of each sample were divided by the mean value of samples from the control group.

### Mitochondrial membrane potential assay

Fluorescent dye Tetramethylrhodamine ethyl ester (TMRE) was used to measure mitochondrial membrane potential (MMP) according to the manufacturer's instructions. H9C2 cells were loaded with TMRE (20 nM) for 30 minutes at 37 °C in the dark. Cells were washed 3 times with PBS, and then fluorescence images were captured using fluorescent microscope (Olympus). In order to determine the relative level of TMRE, values of each sample were divided by the mean value of samples from the control group.

### Measurement of CK-MB release

The level of CK-MB was assayed in serum according to the manufacturer's instructions with a microplate reader (Thermo, China).

### Detecting of myocardium infarct size

Evan blue and triphenyltetrazolium chloride (TTC) double staining were used to assess infarct size. At the end of reperfusion, LAD was ligated again and 0.3 ml 2% Evan blue was injected into the inferior vena cava of mice. After turned to blue, the heart was removed quickly. Then, rinsed with saline, the heart was embedded in OCT and frozen at -20 °C. Five 1mm thick slices were produced and were incubated in 1% TTC at 37 °C for 20 min. The area that turned red after dyeing was defined as area at risk (AAR). The non-ischemic area was deep blue. The area that turned pale was defined as Infarct area (INF). A percent of infarcted area over total area at risk was calculated by Image J software.

### Histopathological analysis of heart

The mouse heart was fixed at least 24 h in 4% formalin solution and embedded in paraffin. The sections (4 µm) were made and fixed on glass slides, then stained with hematoxylin-eosin (H&E). Light microscopy (Olympus) was used to observe the pathological structural changes of heart.

### Propidium iodide staining and Immunofluorescence

We examined the level of apoptosis and necrosis in the myocardium by *in vivo* PI labeling as described previously 23. Briefly, mice were injected with 10 mg/kg PI before sacrifice. Then, myocardium sections of 7 μm were made and stained with Hoechst. For immunofluorescence staining, OCT compound was employed to prepare frozen sections of the hearts and stained for LC3B (1/250 dilution). Fluorescence images were captured using fluorescent microscope (Olympus).Five fields were randomly selected from each sample and used to calculate the cell death rate and the expression level of LC3B.

### Masson staining

The mouse heart were harvested at 7 days after I/R injury and fixed at least 24h in 4% formalin solution and embedded in paraffin. The sections (4 µm) were made and fixed on glass slides. Collagen fibers were stained by Masson's trichrome kit according to the manufacturer protocol.

### Transmission electron microscopy

The myocardium was fixed with 2.5% glutaraldehyde as well as stained in uranyl acetate and dehydrated in ethanol. Following this, they were embedded in epoxy resin and examined with a transmission electron microscope (Hitachi, Tokyo, Japan). Five fields were randomly selected from each sample and used to calculate the number of autophagosomes.

### Protein preparation and Western blotting

Western blotting was carried out in previous studies 24.Tissue or cell lysates were obtained by RIPA lysis Buffer (Beyotime, China). The Protein concentration was measured by BCA kit (Beyotime, China). 20 to 40 mg of protein was loaded for SDS-PAGE before transfered to PVDF membrane (Millipore Corporation, MA, USA). The membranes were blocked by 5% skim milk containing 0.1% Twain 20 for 2h, then washed. After that, the membranes were incubated with primary antibody overnight at 4 °C, then washed and incubated with goat anti-rabbit IgG, peroxidase conjugated (1:10000; Biosharp, China) for 2 hours. The target protein signal was detected and digitalized using ECL by using the ChemiDicTM XRS + Imaging System (Bio-Rad). Signal intensity of the protein was detected using Image J. To determine relative changes in protein expression, signal intensity of the protein was normalized to the control group or sham group which was considered as 1.

### Statistical analysis

Statistical analysis was performed by the SPSS software 21.0. Statistically significant differences were evaluated by Student t test (for two groups) and one-way ANOVA with Tukey's post hoc test or nonparametric Kruskal-Wallis test followed by the Bonferroni test (for multi-group). The data are expressed as mean±SD. P<0.05 was considered significant.

### Materials

Met, CQ, TTC, Evans Blue, dimethyl sulfoxide (DMSO), TMRE, MDC, PI, Hoechst was obtained from Sigma-Aldrich (St. Louis, MO, USA). Miransertib was purchased from Selleck Chemicals (Houston, Texas, USA).DMEM and FBS were purchased from Gibco Laboratories (Life Technologies, Inc., Burlington, ON, Canada).CCK-8 was purchased from Dojindo Laboratories (Tokyo, Japan).The commercial assay kits for the CK-MB were from Jiancheng Bioengineering (Nanjing, Jiangsu, China). The primary antibodies used were anti-IL-1β, anti-glyceraldehyde-3-phosphate dehydrogenase (GAPDH) Bioworld (MN, USA), anti-IL-6 (Abcam, Cambridge, MA, USA), anti-Bax, anti-phospho-Akt, anti-Akt, anti-phospho-mTOR and mTOR, anti-phospho-AMPK and AMPK, anti-Beclin-1, anti-P62, anti-Atg5 were purchased from Cell Signaling Technology (Beverly, MA, USA). In addition, anti-LC3B from Sigma and anti-Bcl-2 from Absin were used. All the antibodies were from rabbits and dilutions of antibodies were 1:1000, except anti-GAPDH (1:5000).

## Results

### Met protects heart from I/R injury by reducing inflammation and cell apoptosis

**Figure 1:**
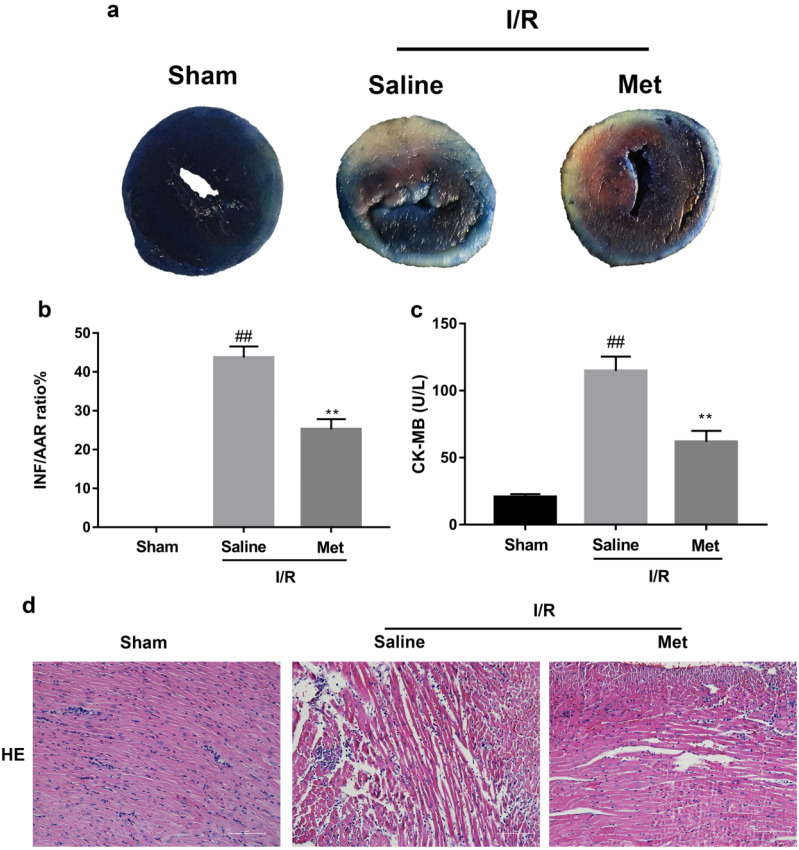

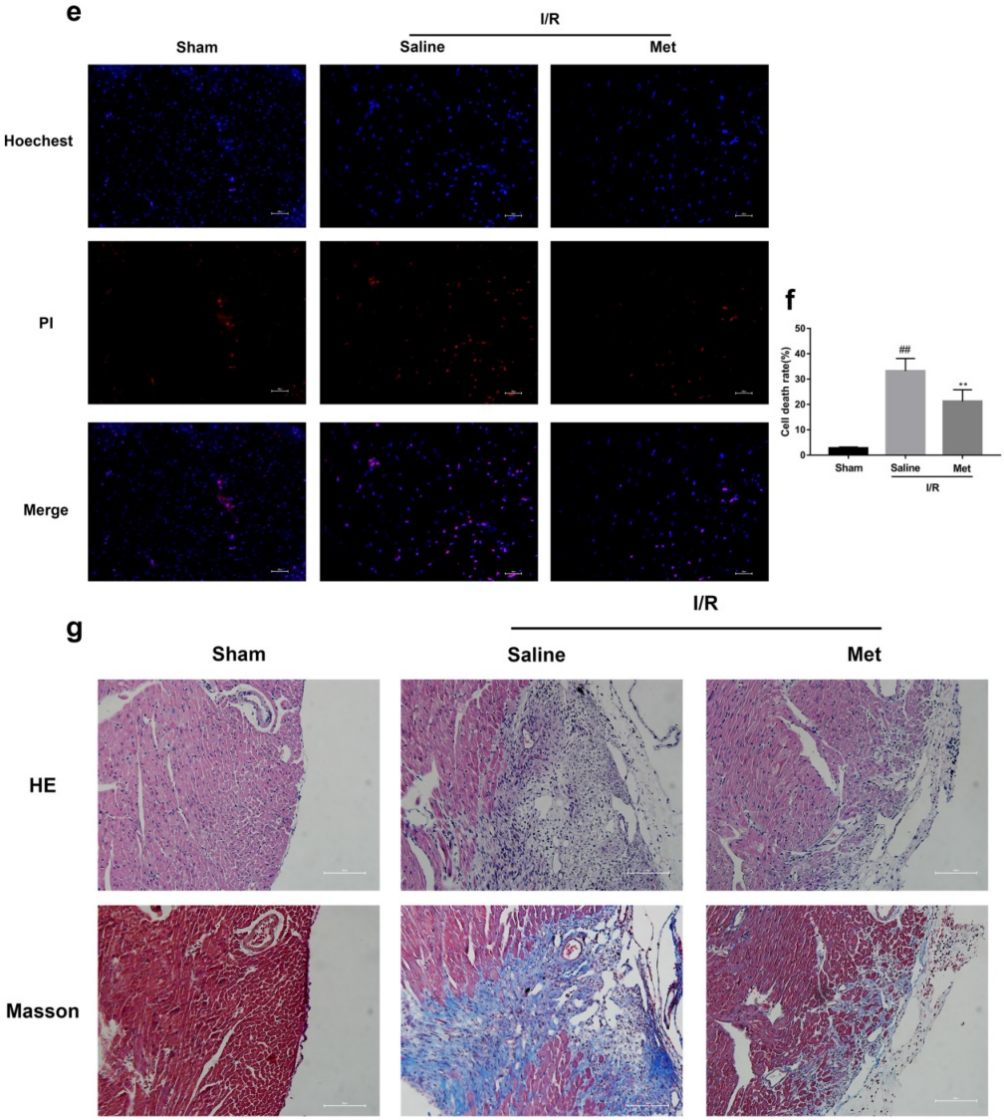

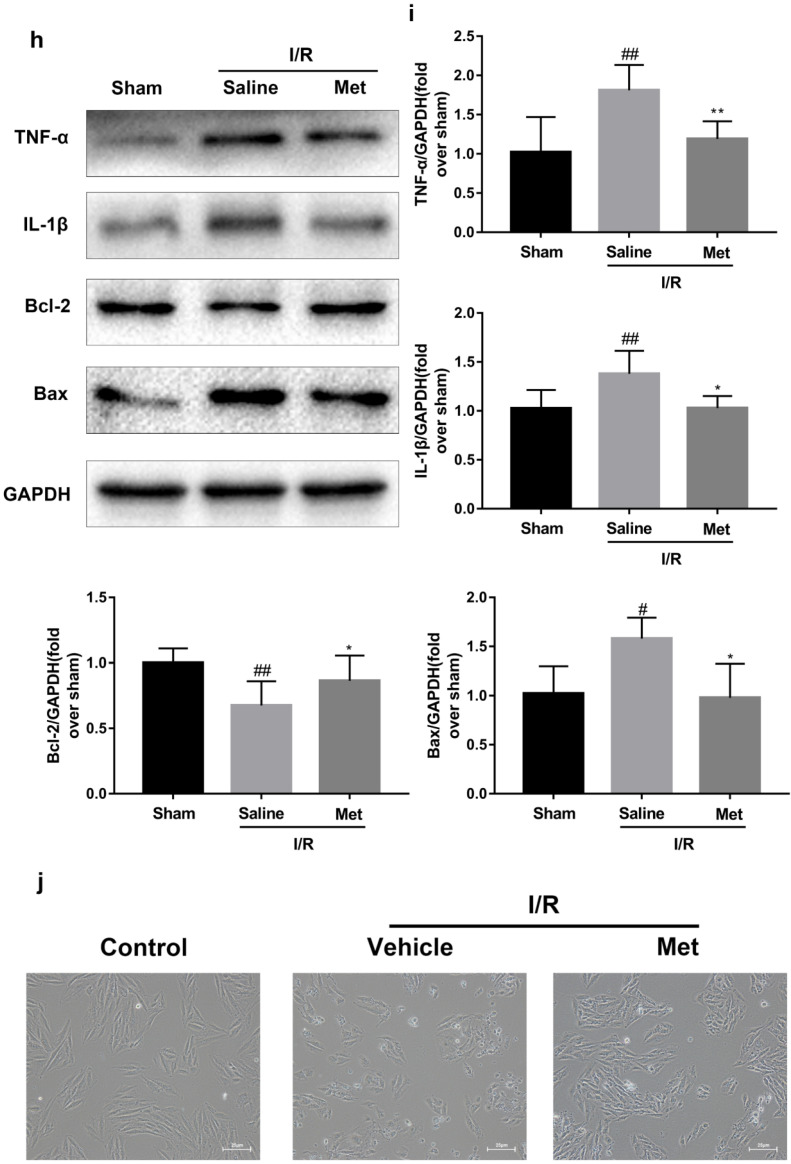

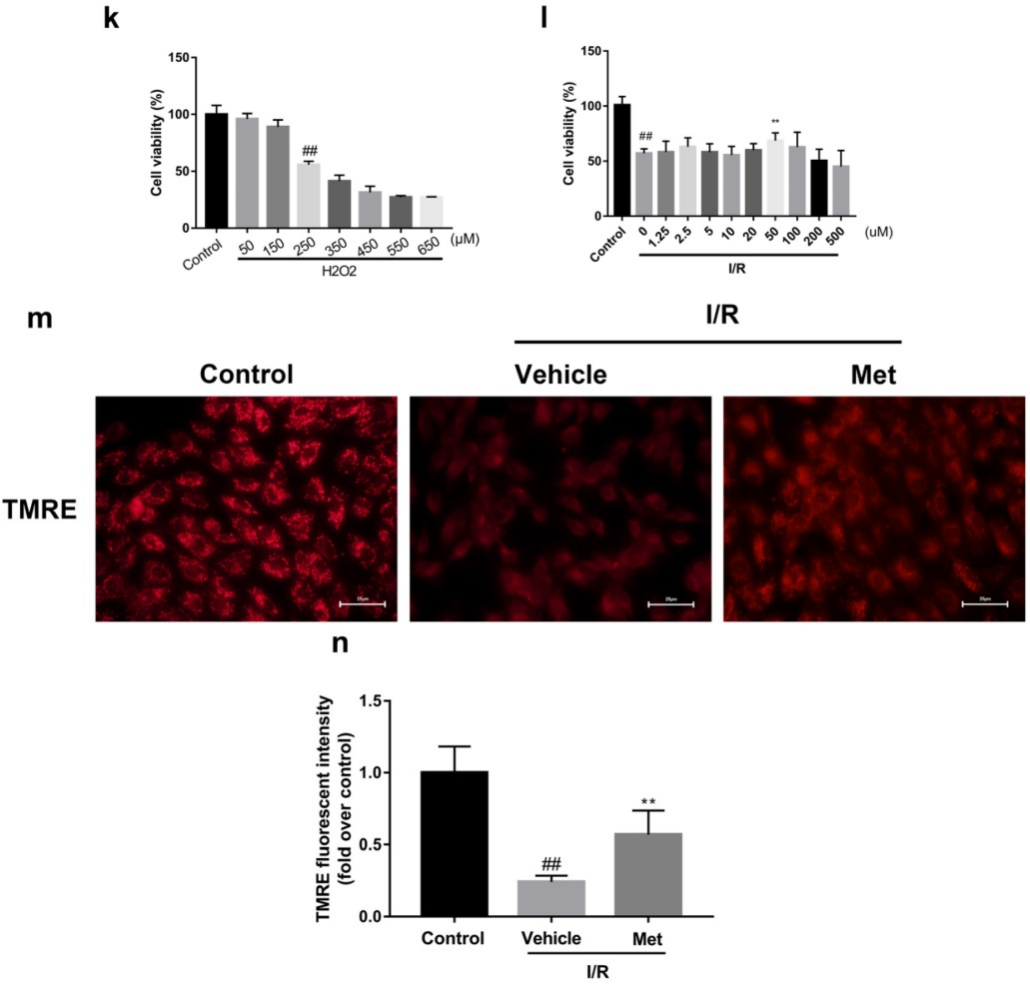

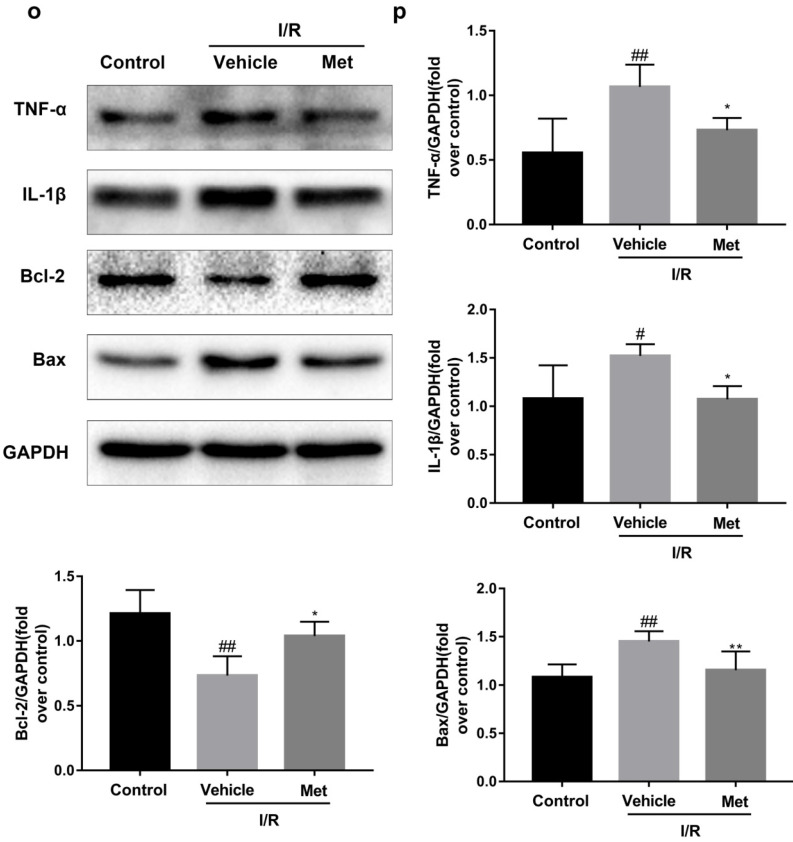
** Met attenuates I/R injury.** Mice were treated with Met (125 µg/kg;i.v.) 15 min prior to ischemia, (**a, b**)TTC stain, (**c**)CK-MB release were analyzed after reperfusion for 4h. (**d**) HE stain sections, (**e, f**) PI stain were analyzed after reperfusion for 24h. (**g**)HE stain and Masson stain sections were analyzed after reperfusion for 7 days. (**h,i**) After reperfusion for 4h, western blot results of TNF-α,IL-1β,Bcl-2 and Bax in the sham group, I/R group and Met pretreatment group were analyzed. (**k**) H9C2 cells were treated with various concentrations of H_2_O_2_ (50, 150, 250, 350, 450, 550,or 650 µM) for 4h,and cell viability was measured. (**l**) H9C2 cells were pretreated with Met (1.25, 2.5, 5, 10, 20, 50, 100, 200,or 500 µM) for 12h and then subjected to I/R injury, and cell viability was measured. (**j**) H9C2 cells were pretreated with Met (50 µM) then subjected to I/R injury and morphological changes of cells was analyzed. Scale bar: 25 µm. (**m, n**) Representative immunofluorescence images of H9C2 loaded with TMRE. The MMP of cells was determined. Scale bar: 25 µm. (**o, p**) After I/R injury, western blot results of TNF-α,IL-1β,Bcl-2 and Bax in the control group, I/R group and Met pretreatment group were analyzed. n = 6. Values are expressed as the means ± SD. #p<0.05, ##p<0.01 vs. the sham or control group, *p<0.05, **p<0.01 vs. IR group, ^p<0.05, ^^p<0.01 vs. IR+Met group.

The mice were injected with Met (125 µg/kg, i.v.) 15 min before surgery, then the myocardial infarction size, serum CK-MB, myocardial apoptosis as well as myocardial pathological changes were detected. As shown in Figure 1a to 1c, Met was able to reduce infarct size and the level of CK-MB following I/R injury. HE staining showed that I/R injury induced large numbers of ruptured myocardial fibers, myocardial necrosis and inflammatory cell infiltration; however, metformin prevented myocardial damage (Figure 1d). Besides, PI staining also indicated that pretreatment with Met attenuated cell apoptosis and necrosis (Figure 1e and 1f). A week after I/R injury, a large number of myocardial necrosis (HE staining) and collagen deposition (Masson staining) were detected in the infarcted area. However, a single acute administration of Met could alleviate such long-term adverse effects induced by I/R injury (Figure 1g). As shown in Figure 1h and 1i, I/R injury significantly decreased Bcl-2, an anti-apoptotic protein, but upregulated Bax, a pro-apoptotic protein, as well as proinflammatory cytokines such as TNF-α and IL-1β. However, Met pretreatment reversed these effects following reperfusion for 4h. In order to further confirm the protective effect of Met, we designed an *in vitro* experiment. By detecting cell viability, we pretreated H9C2 cells with different concentrations of H2O2 for 4 hours, and finally decided to treat H9C2 cells with 250 µM H2O2 for 4 hours to mimic I / R injury *in vivo* (Figure 1k). Similarly, we found that pretreatment with 50µM Met for 12 hours had the greatest protective effect on I/R injury, and 50µM Met was used in the subsequent cell experiments (Figure 1j and 1i). In addition, I/R injury decreases the fluorescence intensity of TMRE, which is closely related to cell death and apoptosis 25, 26, however, Met was able to prevent this phenomenon (Figure 1m). Consistent with *in vivo* results, Met reduced the expression of I/R-induced apoptosis and inflammation-related proteins in H9C2 cells (Figure 1o and 1p).

### Met inhibits autophagosomes formation and restores I/R-impaired autophagosome processing

**Figure 2:**
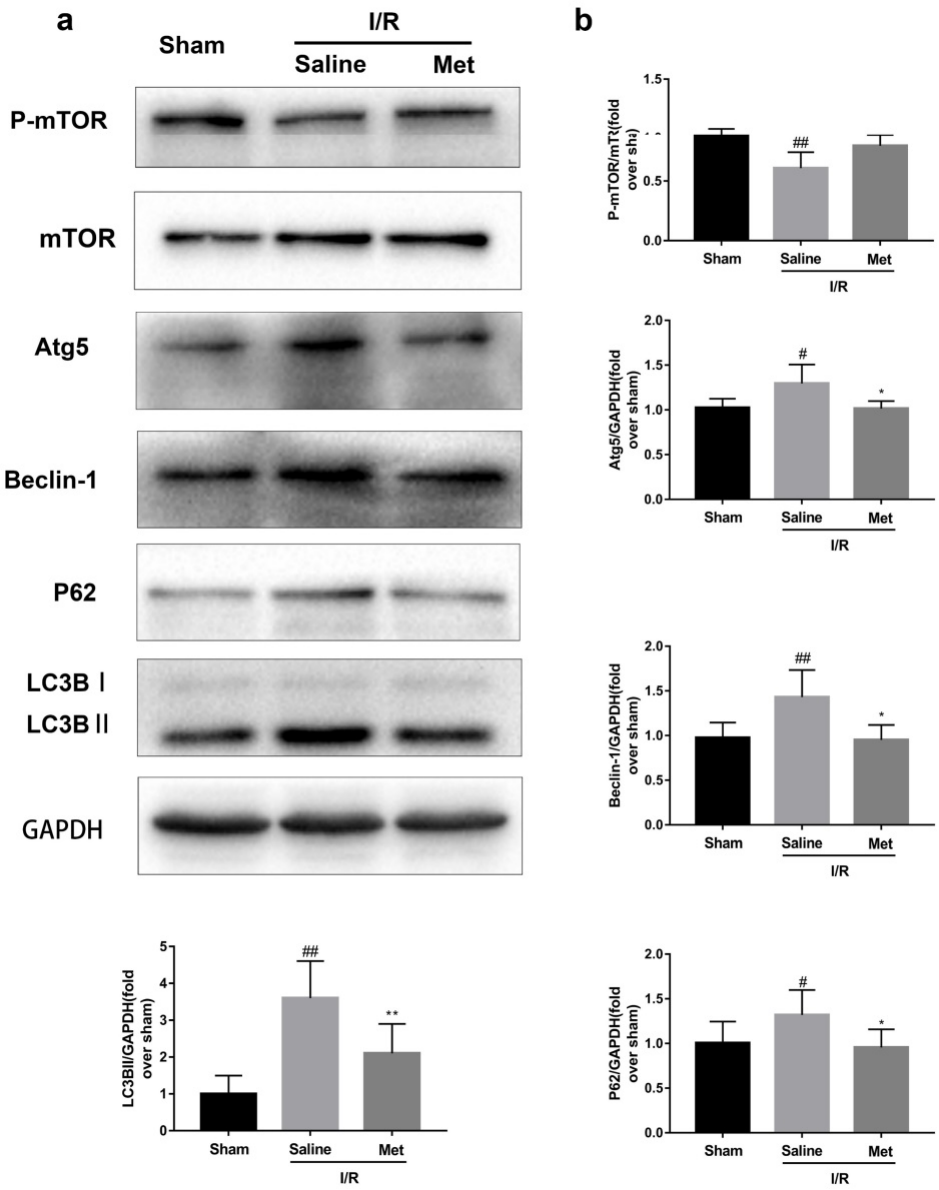

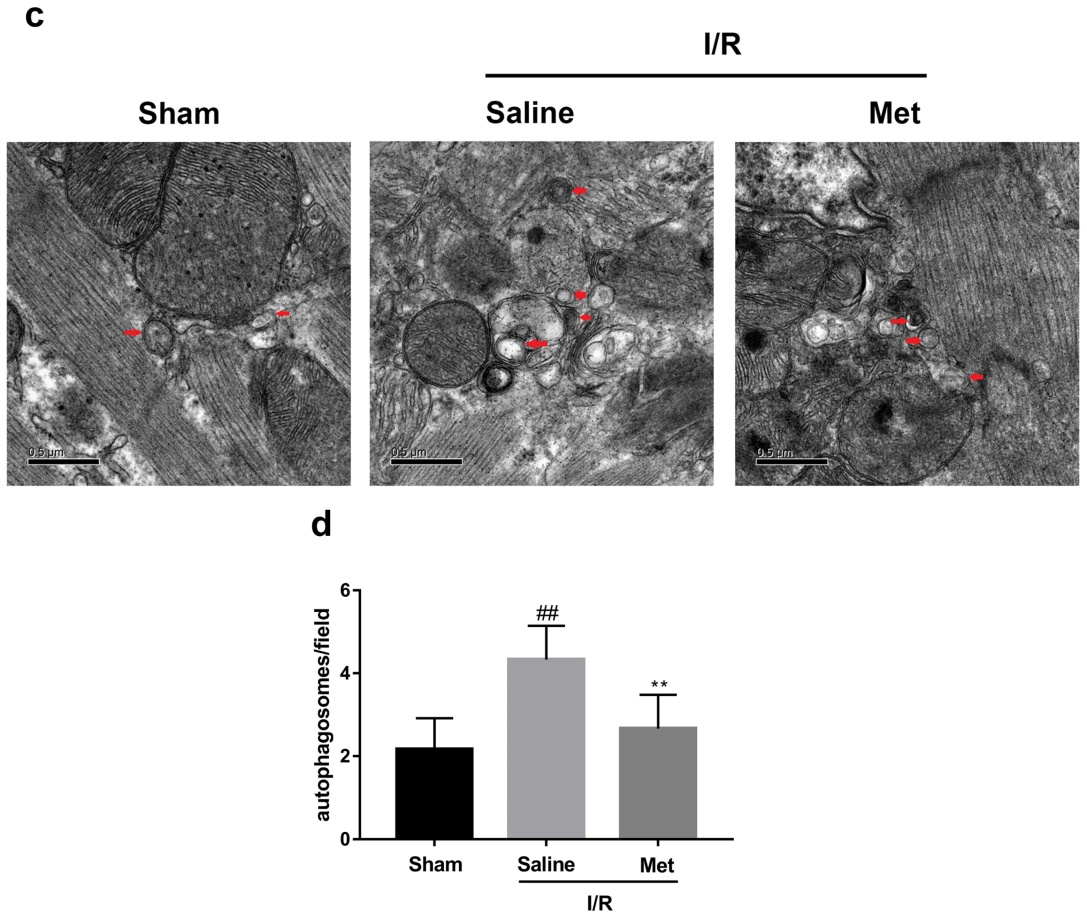

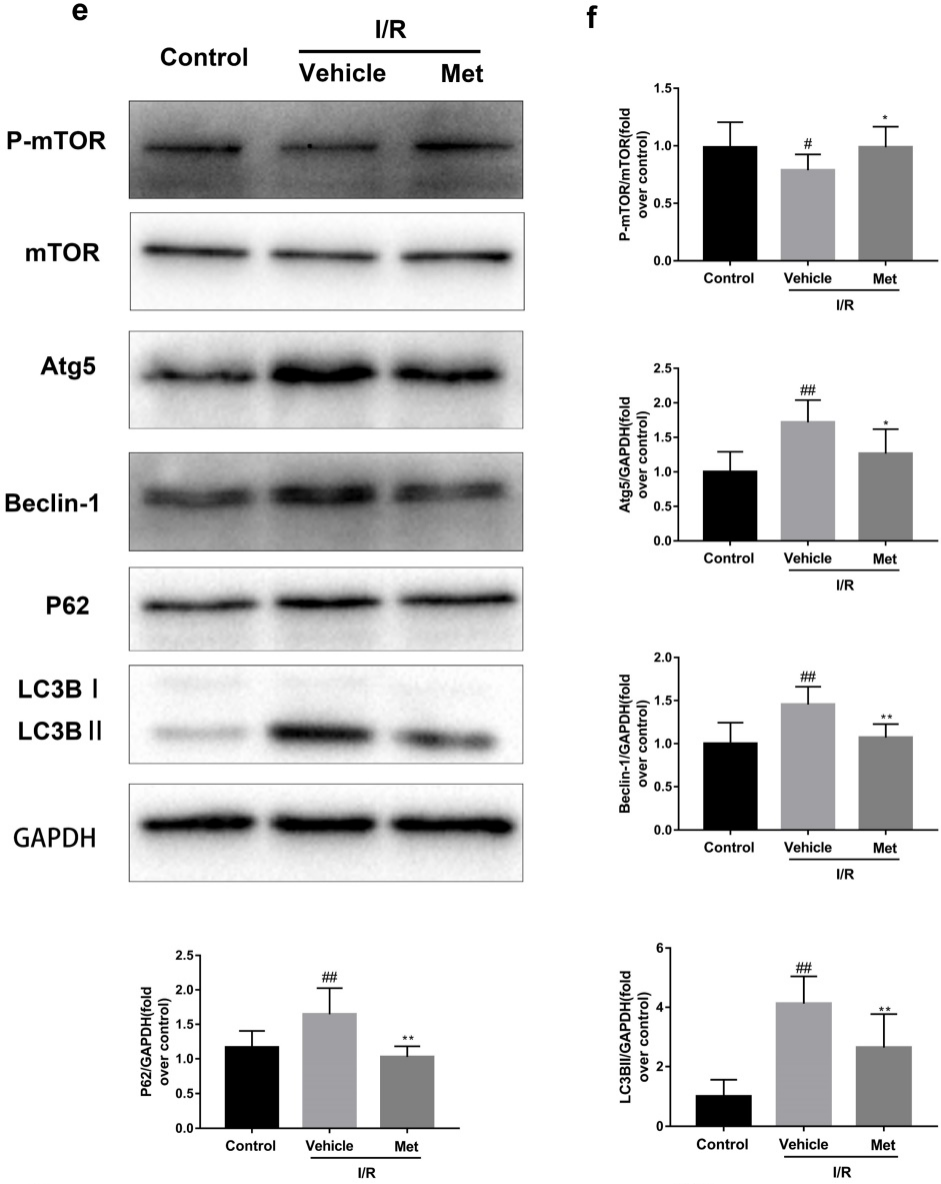

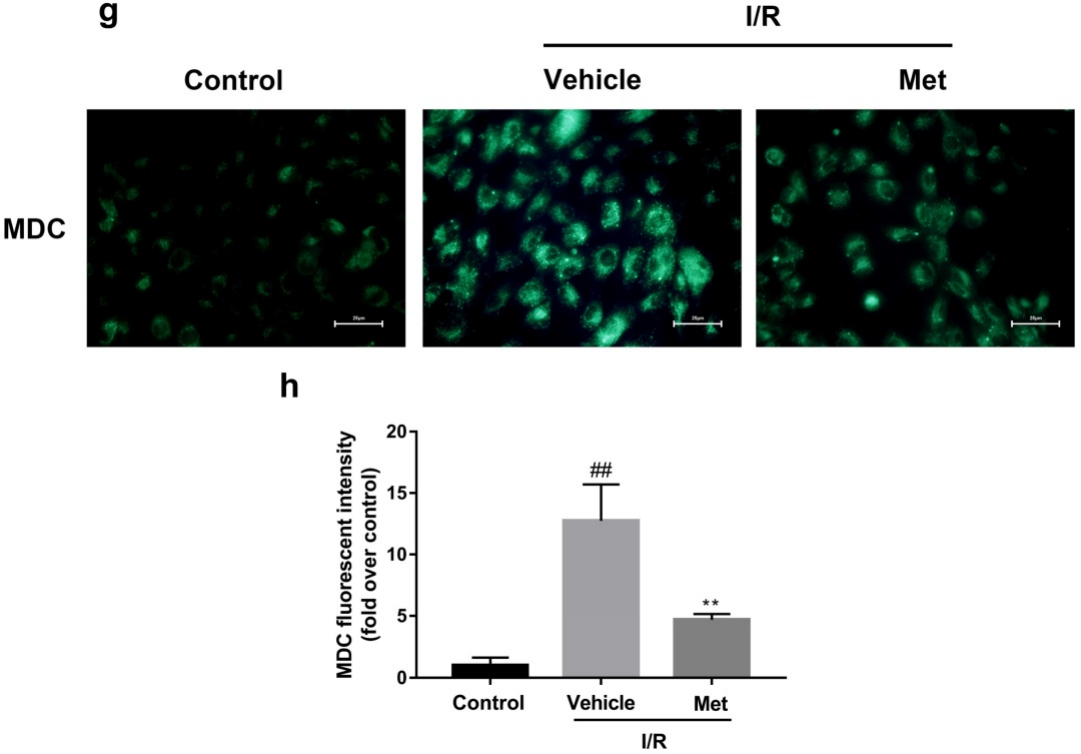
** Met inhibits autophagosomes formation and restores autophagosome processing during I/R injury.** In I/R induced mice hearts after various treatment, (**a, b**) protein expression levels of P-mTOR/ mTOR ,Atg5, Beclin-1, LC3B and P62 were analyzed by western blot. (**c, d**) Autophagosomes in mice myocardium at 4h of reperfusion were detected by transmission electron micrographs. Autophagosomes are indicated by red arrows. Scale bar: 0.5 µm. After H9C2 cells were pretreated with Met and suffered from I/R injury, (e**, f**) protein expression levels of P-mTOR/ mTOR, Atg5, Beclin-1, LC3B and P62 were analyzed by western blot. (**g, h**) Representative immunofluorescence images of H9C2 loaded with MDC. The autophagosomes of cells was determined. Scale bar: 25 µm. n = 6. Values are expressed as the means ± SD. #p<0.05, ##p<0.01vs. the sham or control group, *p<0.05, **p<0.01 vs. IR group, ^p<0.05, ^^p<0.01 vs. IR+Met group.

Studies have reported that mTOR, Atg5 and Beclin-1 are involved in the formation process of autophagosomes 27. P62, a marker of autophagosome processing, can take ubiquitinated aggregates into autophagosomes and be degraded along with the autophagic process 28. LC3BII is considered as a classical marker of autophagy activity 21. Both mice hearts (Figure 2a and 2b) and H9C2 cells (Figure 2e and 2f) significantly increased the Atg5, Beclin-1, LC3BII and P62 abundance, but decreased the phosphorylation of mTOR during I/R injury, while it was reversed by Met. It indicated that Met attenuated autophagosomes formation and restored autophagosome processing which was induced by I/R injury. Furthermore, observation of autophagy activity by TEM revealed that Met preconditioning reduced the number of autophagosomes in myocardium after I/R injury (Figure 2c and 2d). Similar to the *in vivo* results, the accumulation of autophagic vesicles detected by MDC showed that the number of autophagic vacuoles in Met treatment group was significantly less than that in I/R injury group (Figure 2g and 2h).

### Inhibition of autophagosomes formation accounts for Met-mediated anti-inflammatory and anti-apoptotic effects in myocardial I/R injury

**Figure 3:**
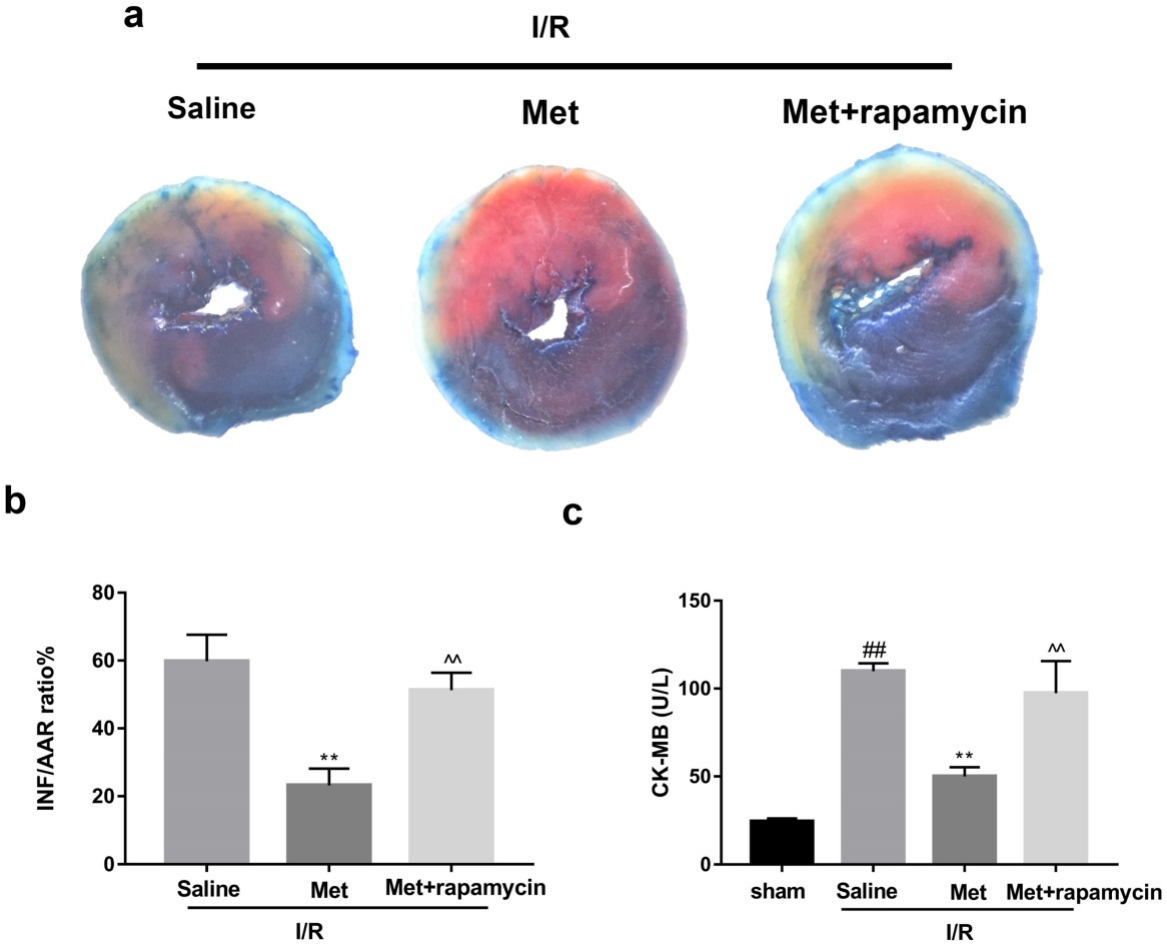

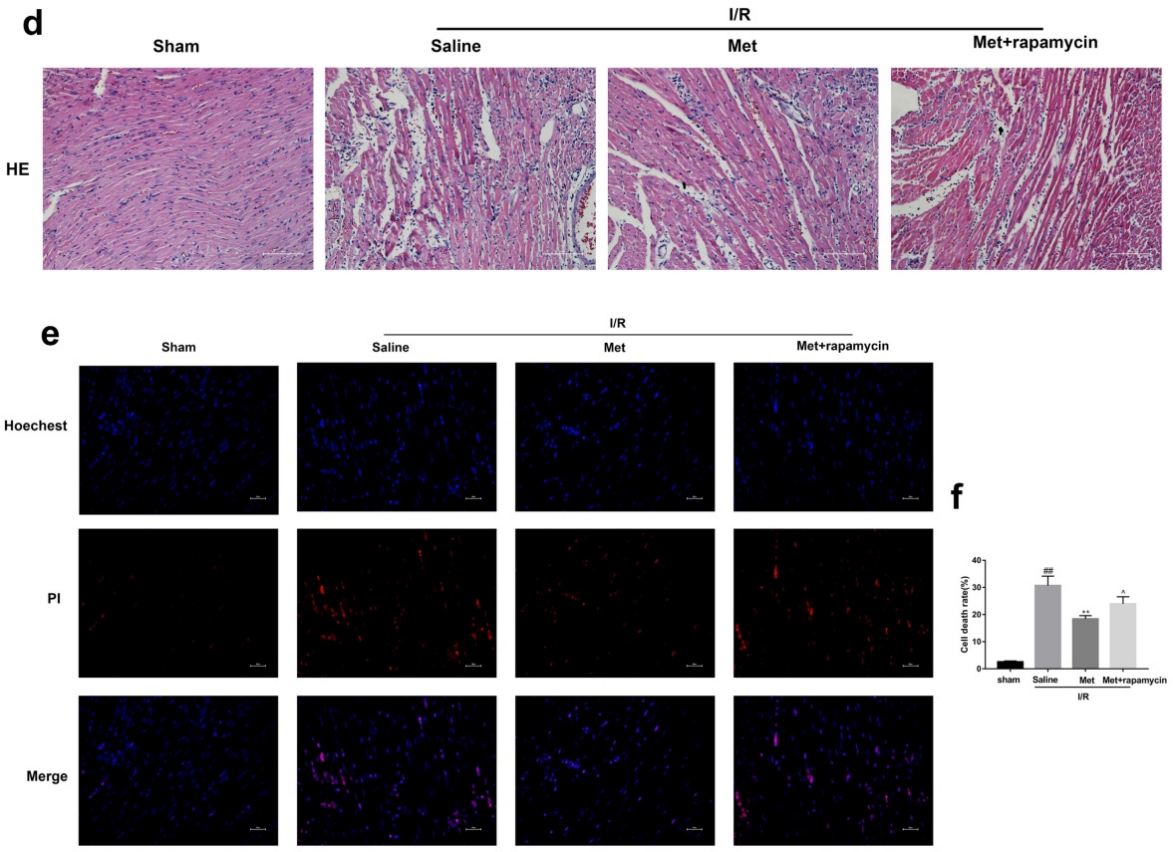

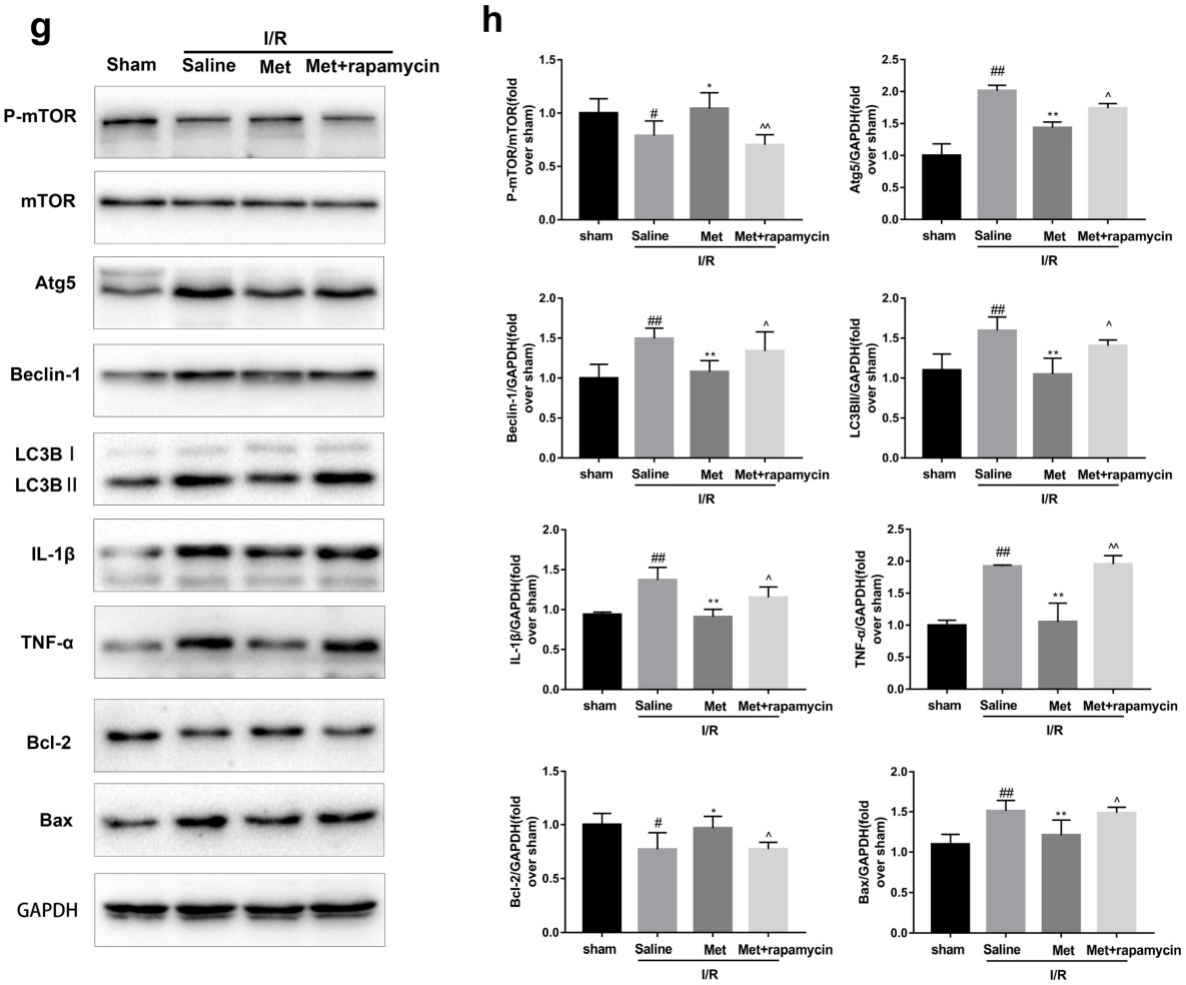

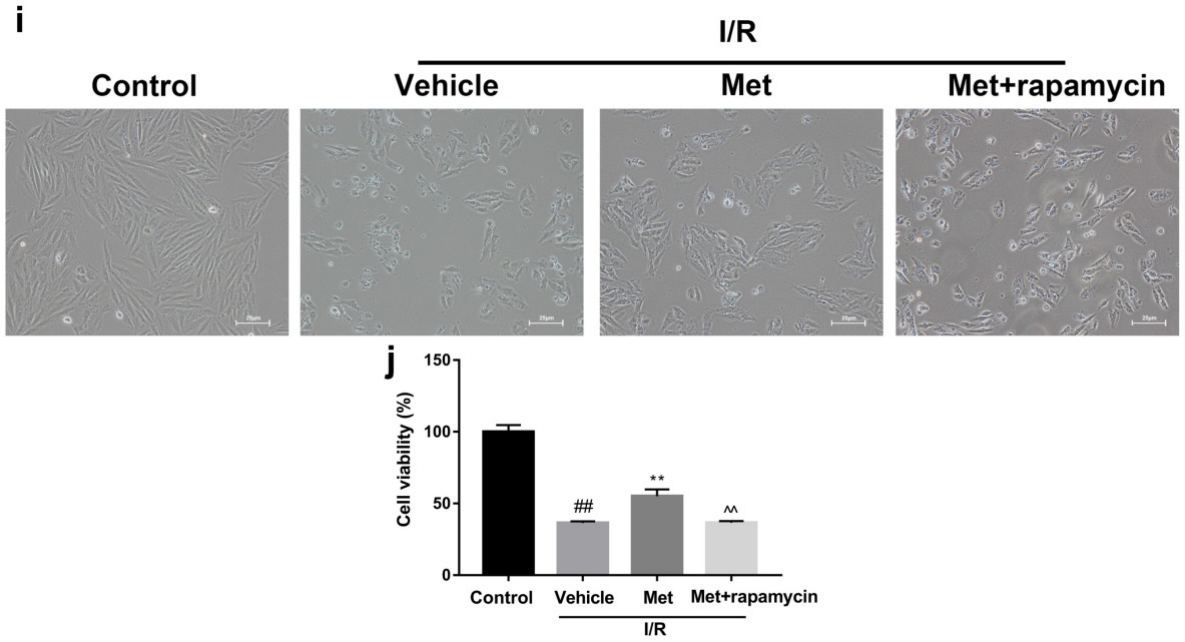

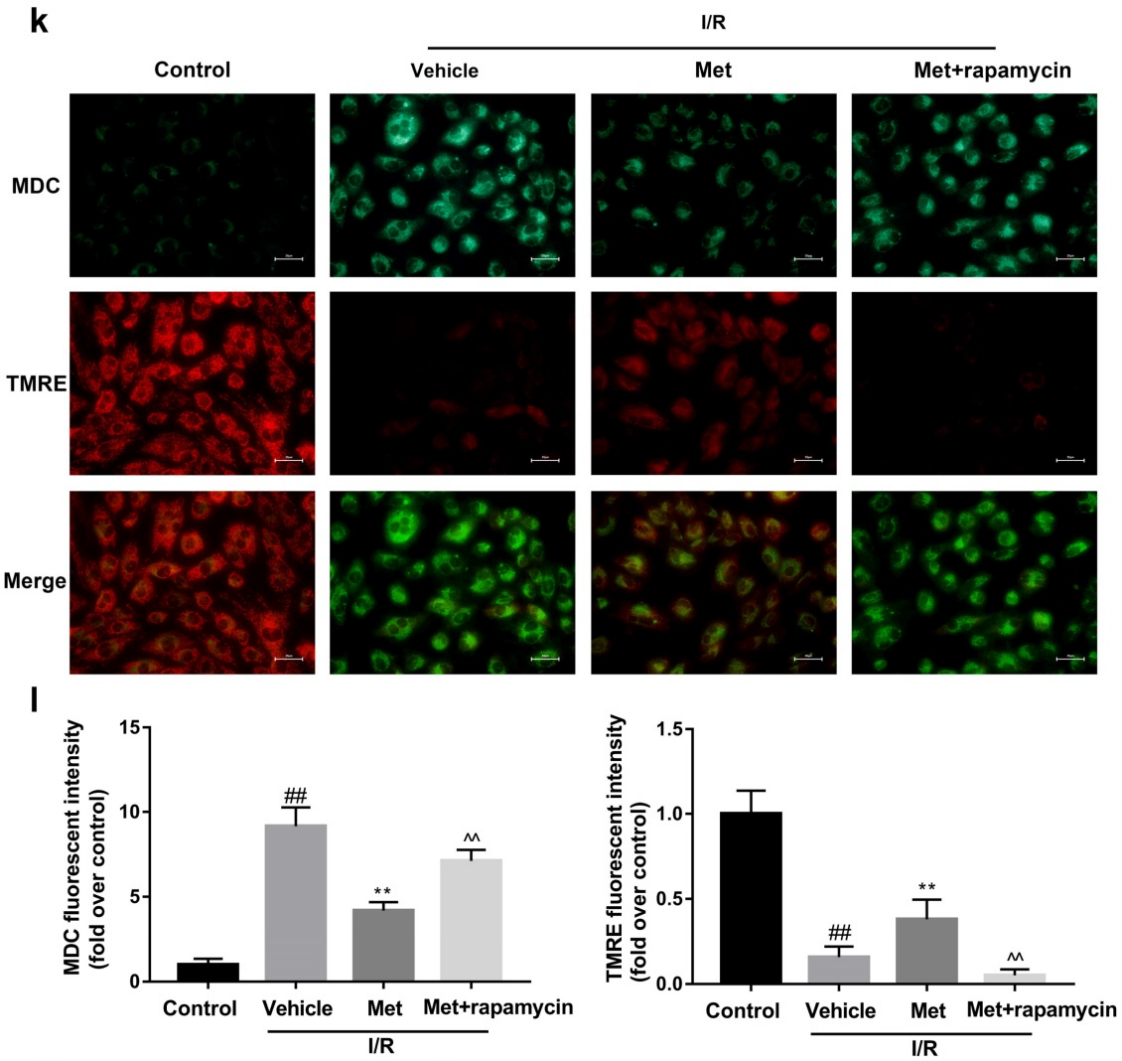

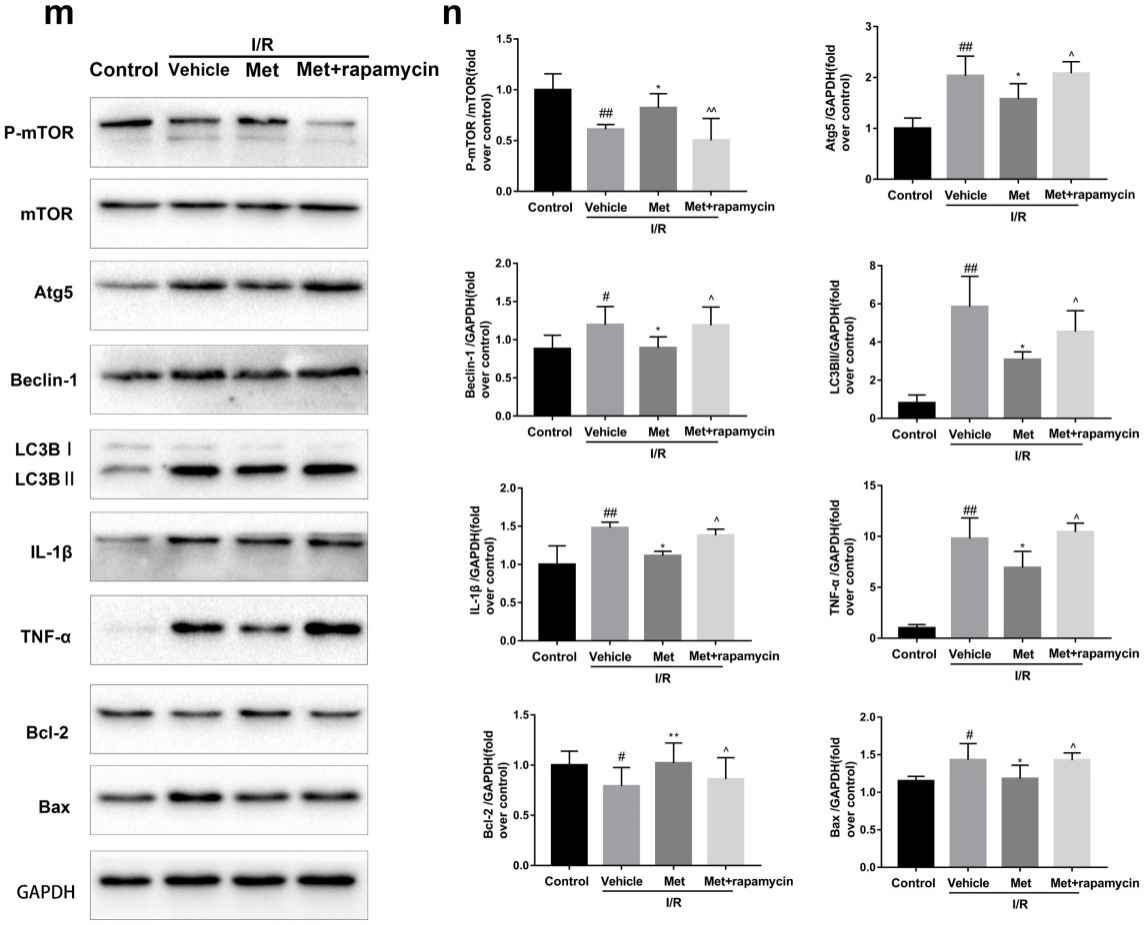
** Met attenuated I/R injury via inhibition of autophagosomes formation.** Mice were pretreated with rapamycin (0.25 mg/kg; i.p.)1h before ischemia and Met (125 µg/kg; i.v.) 15 min prior to ischemia, then killed at 4h after reperfusion for detection of (**a, b**) TTC stain, (**c**) Serum CK-MB levels, (**e, f**) PI stain, scale bar: 25 µm. (**g, h**) Western blot indicating expression of P-mTOR/ mTOR, Atg5, Beclin-1, LC3B, TNF-α, IL-1β, Bcl-2 and Bax proteins. The mice were killed at 24h after reperfusion for detection (**d**) Representative HE stained sections, scale bar:25 µm. After H9C2 cells were pretreated with Met (50 µM) for 12h and rapamycin (100 nM) for 2h then suffered from I/R injury for detection of (**i, j**) cell viability, scale bar: 25 µm. (**k, l**) Double immunofluorescence of MDC and TMRE in H9C2 cells. (Green signal represents MDC, red signal represents TMRE) scale bar: 25 µm, (m, n)Western blot indicating expression of P-mTOR/ mTOR, Atg5, Beclin-1, LC3B, TNF-α, IL-1β, Bcl-2 and Bax proteins. n = 6. Values are expressed as the means ± SD. #p<0.05, ##p<0.01 vs. the sham or control group, *p<0.05, **p<0.01 vs. IR group, ^p<0.05, ^^p<0.01 vs. IR+Met group.

To investigate whether the protective effect of Met is related to inhibition of autophagosomes formation, we used a classical autophagy activator rapamycin which inhibited mTOR to induce formation of autophagosomes. As shown in Figure 3a to 3c, inducing of autophagosomes formation by rapamycin in Met pretreated group remarkably reversed effects of Met on infarct size and serum CK-MB level. Similarity, HE staining and PI staining showed rapamycin aggravated myocardial damage as well as myocardial apoptosis, necrosis and inflammatory cell infiltration after I/R (Figure 3d-f). In addition, MDC and TMRE double-labeled staining results showed that accumulation of autophagosomes was reduced and the fluorescence intensity of TMRE was increased by Met, however, rapamycin reversed this effect (Figure 3k and 3l). This is consistent with cell morphological changes and cell viability test (Figure 3i and 3j). In line with the findings above, both *in vivo* and *in vitro*, the marker protein of autophagosomes formation such as P-mTOR/ mTOR, Atg5 and Beclin-1 as well as LC3BII were increased following treating with rapamycin, however, inflammatory related proteins (TNF-α and IL-β) and apoptotic protein (Bax) increased, while anti-apoptotic protein (Bcl-2) decreased(Figure 3g and 3h, 3m and 3n). These results indicated that inhibition of autophagosomes formation by Met could protect myocardial from apoptosis and inflammation.

### Met inhibits I/R-induced apoptosis and inflammation via restoration of autophagosome processing

**Figure 4:**
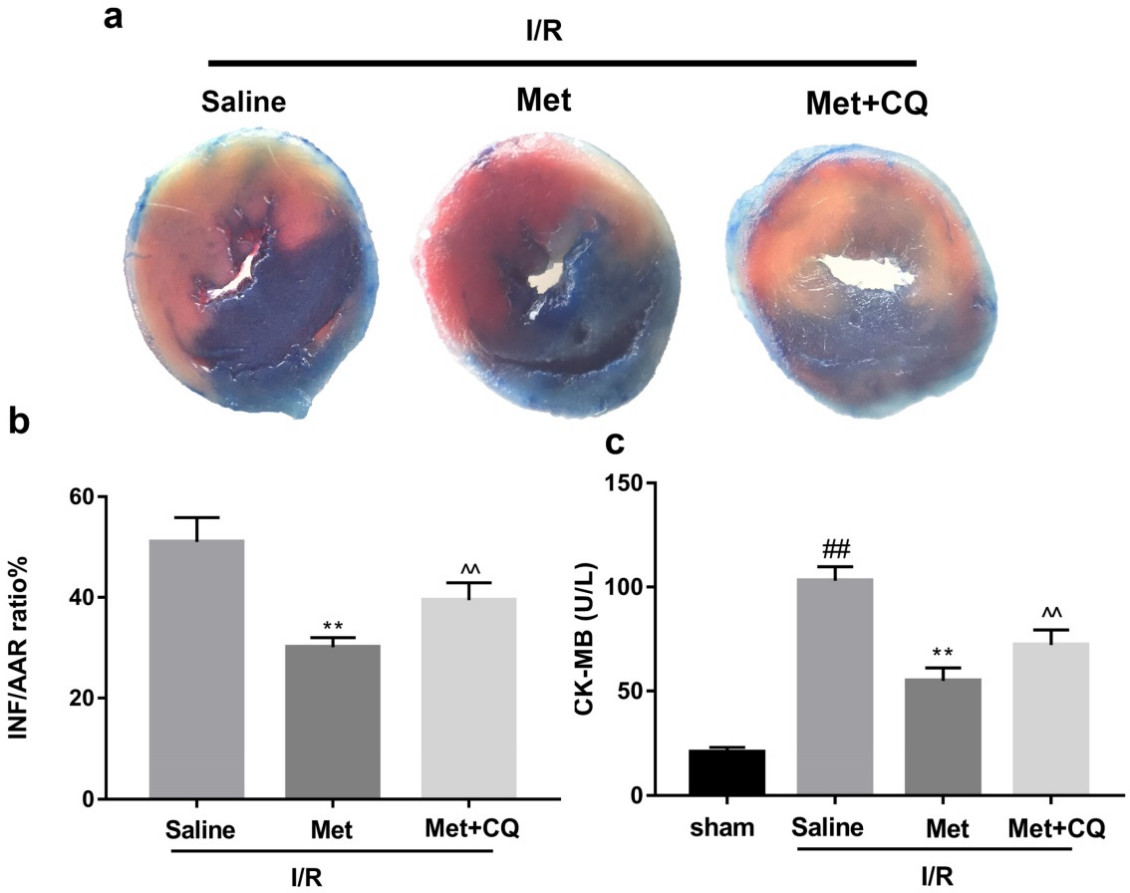

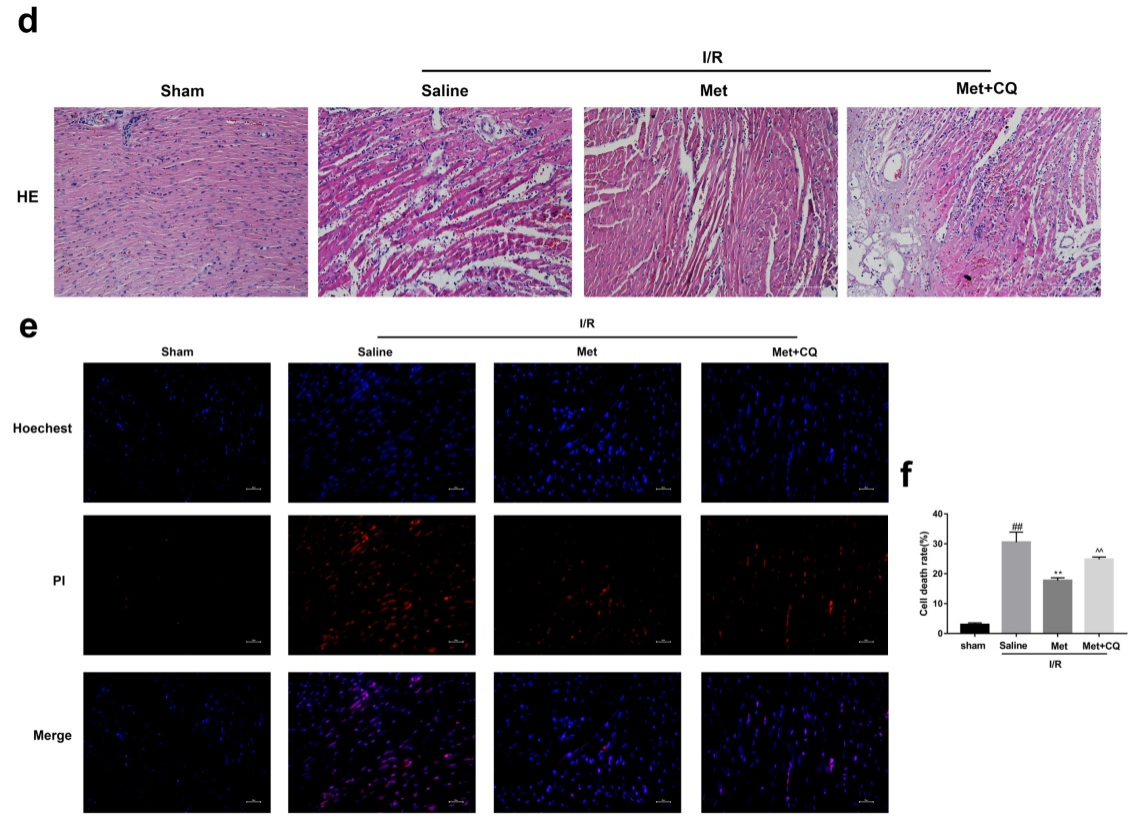

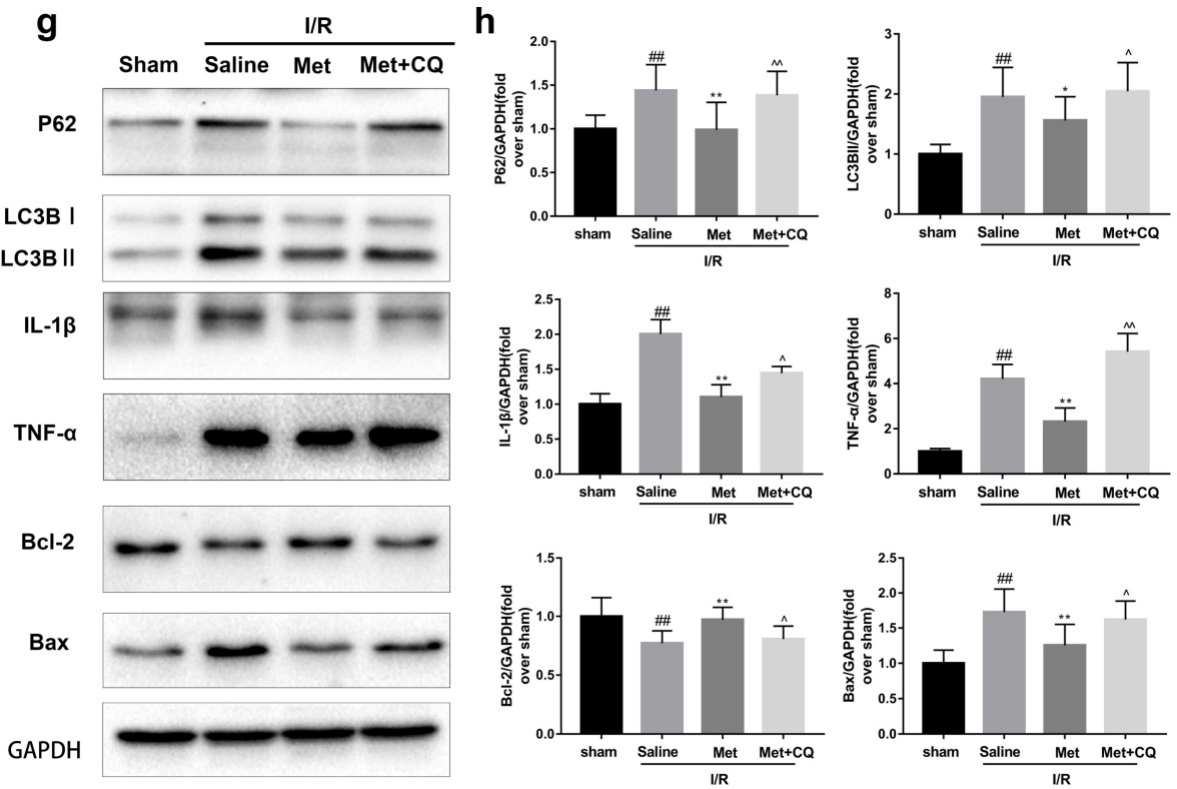

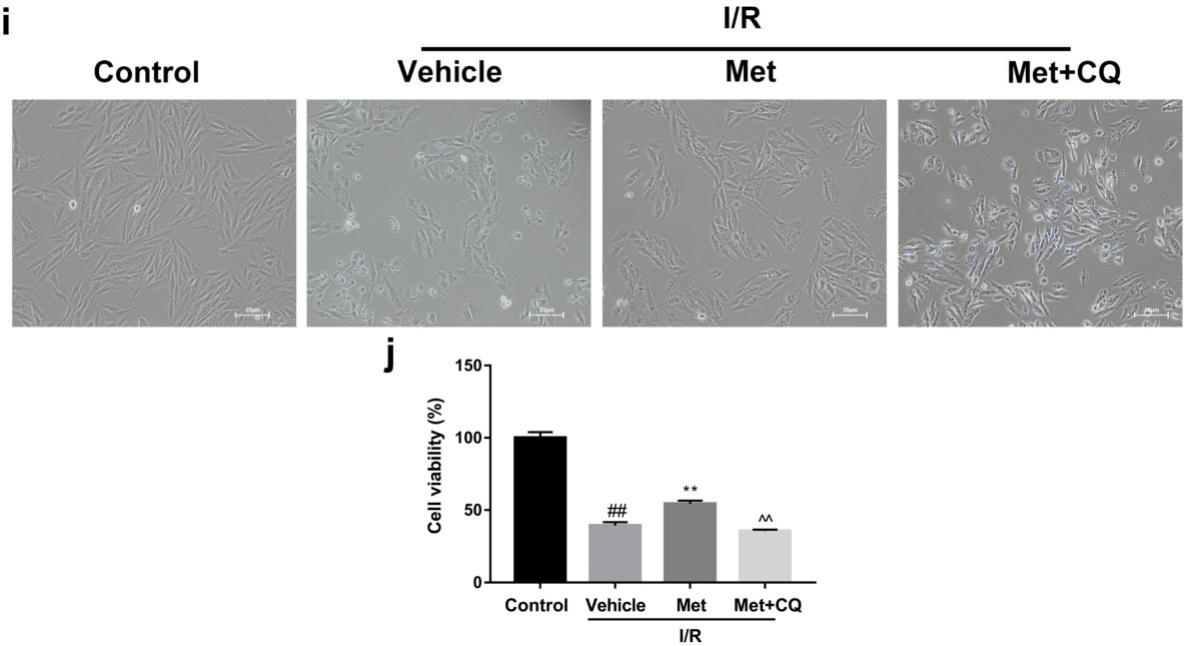

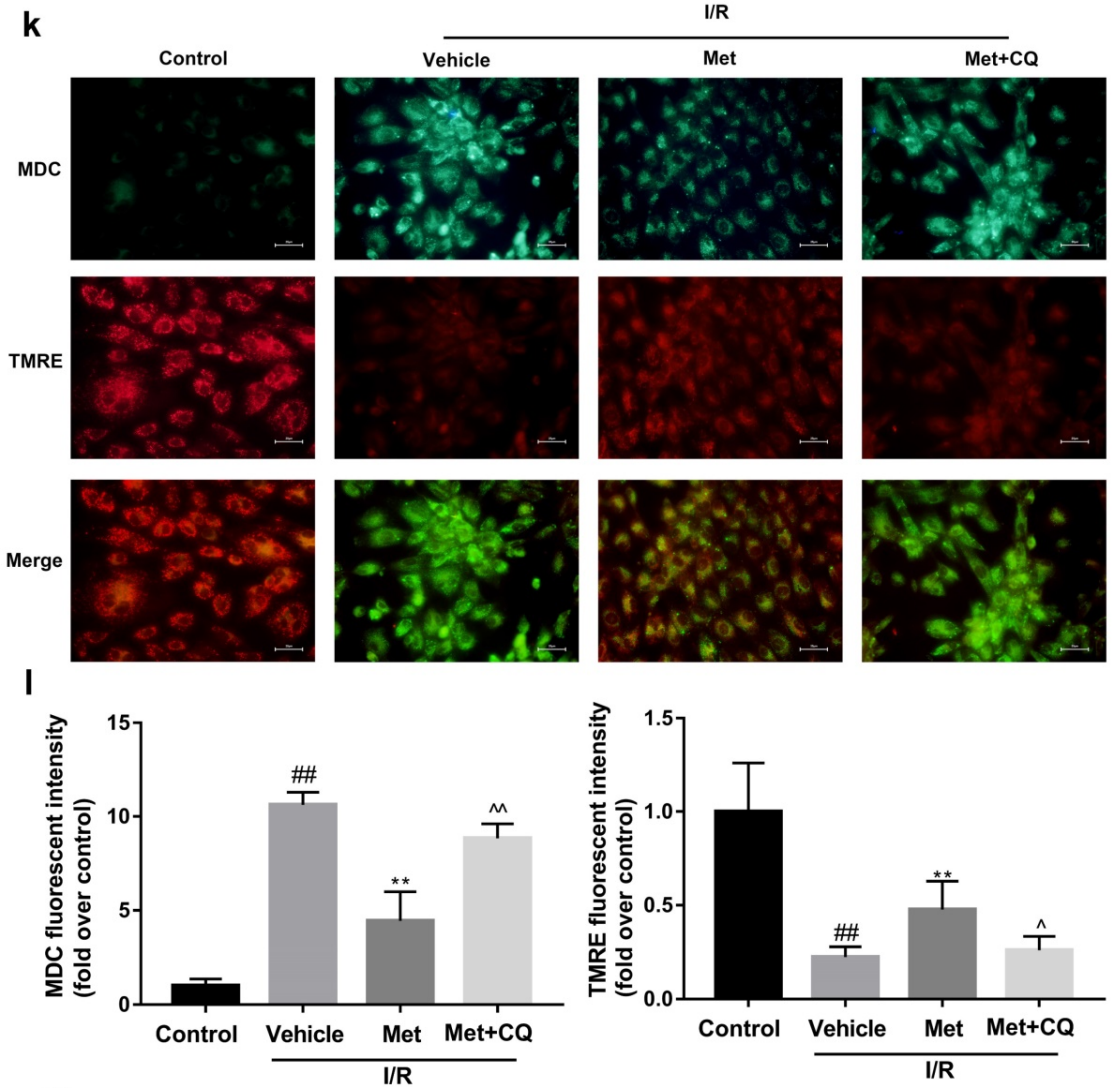

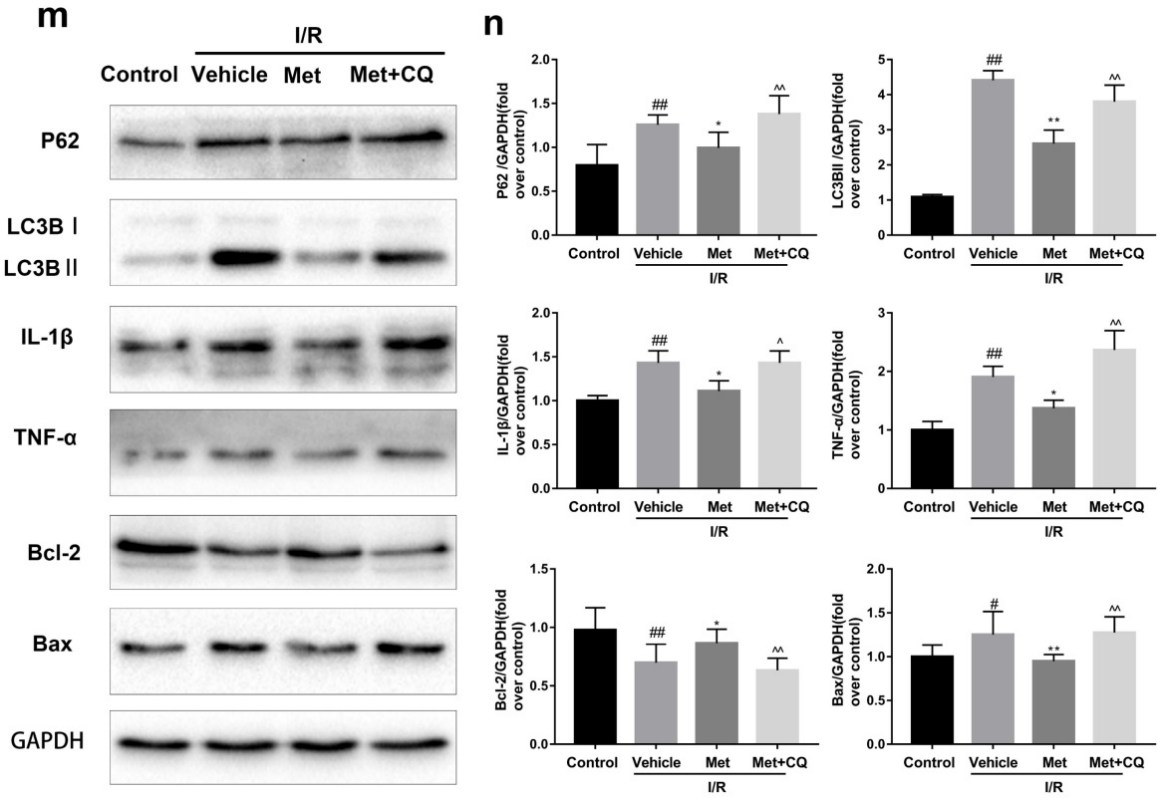
** Met attenuated I/R injury via restores of autophagosome processing.** Mice were pretreated with CQ (10 mg/kg; i.p.)1h before ischemia and Met (125 µg/kg; i.v.) 15 min prior to ischemia, then killed at 4h after reperfusion for detection of (**a, b**) TTC stain, (**c**) Serum CK-MB levels, (**e, f**) PI stain, scale bar: 25 µm. (**g, h**) Western blot indicating expression of P-mTOR/ mTOR, Atg5, Beclin-1, LC3B, TNF-α, IL-1β, Bcl-2 and Bax proteins. The mice were killed at 24h after reperfusion for detection of (**d**) Representative HE stained sections, scale bar: 25 µm. After H9C2 cells were pretreated with Met (50 µM) and CQ (10 µM) for 12h then suffered from I/R injury for detection of (**i, j**) cell viability, scale bar: 25 µm. (**k, l**) Double immunofluorescence of MDC and TMRE in H9C2 cells. (Green signal represents MDC, red signal represents TMRE) scalebar: 25 µm. (**m, n**) Western blot indicating expression of P-mTOR/ mTOR, Atg5, Beclin-1, LC3B, TNF-α, IL-1β, Bcl-2 and Bax proteins, n = 6. Values are expressed as the means ± SD. #p<0.05, ##p<0.01 vs. the sham or control group, *p<0.05, **p<0.01vs. IR group, ^p<0.05, ^^p<0.01 vs. IR+Met group.

To identify whether restoration of autophagosome processing was involved in the cardioprotective of metformin, CQ was used to block the processing. By analyzing WB results of p62 and LC3BII both *in vivo* and *in vitro*, we found that CQ successfully inhibited the fusion of autophagosomes with lysosomes. At the same time, the expression of TNF-α, IL-β and Bax was upregulated but Bcl-2 was decreased (Figure 4g and 4h, 4m and 4n). As shown in Figure 4a-f, the protective effect of Met on myocardium was significantly suppressed by CQ, with a higher level of infarct size, serum CK-MB, ruptured myocardial fibers, myocardial apoptosis and necrosis as well as inflammatory cell infiltration. Correspondingly, destruction of autophagosome processing by CQ resulted in H9C2 cells of Met group more sensitive to I/R induced cell injury as indicated by decreased cell viability, cell morphological damage and lower fluorescence intensity of TMRE with higher fluorescence intensity of MDC (Figure 4i-l). These results demonstrated that restoration of autophagosome processing was involved in Met-mediated cardioprotection effect.

### Activation of Akt was involved in Met treatment on I/R injury

**Figure 5:**
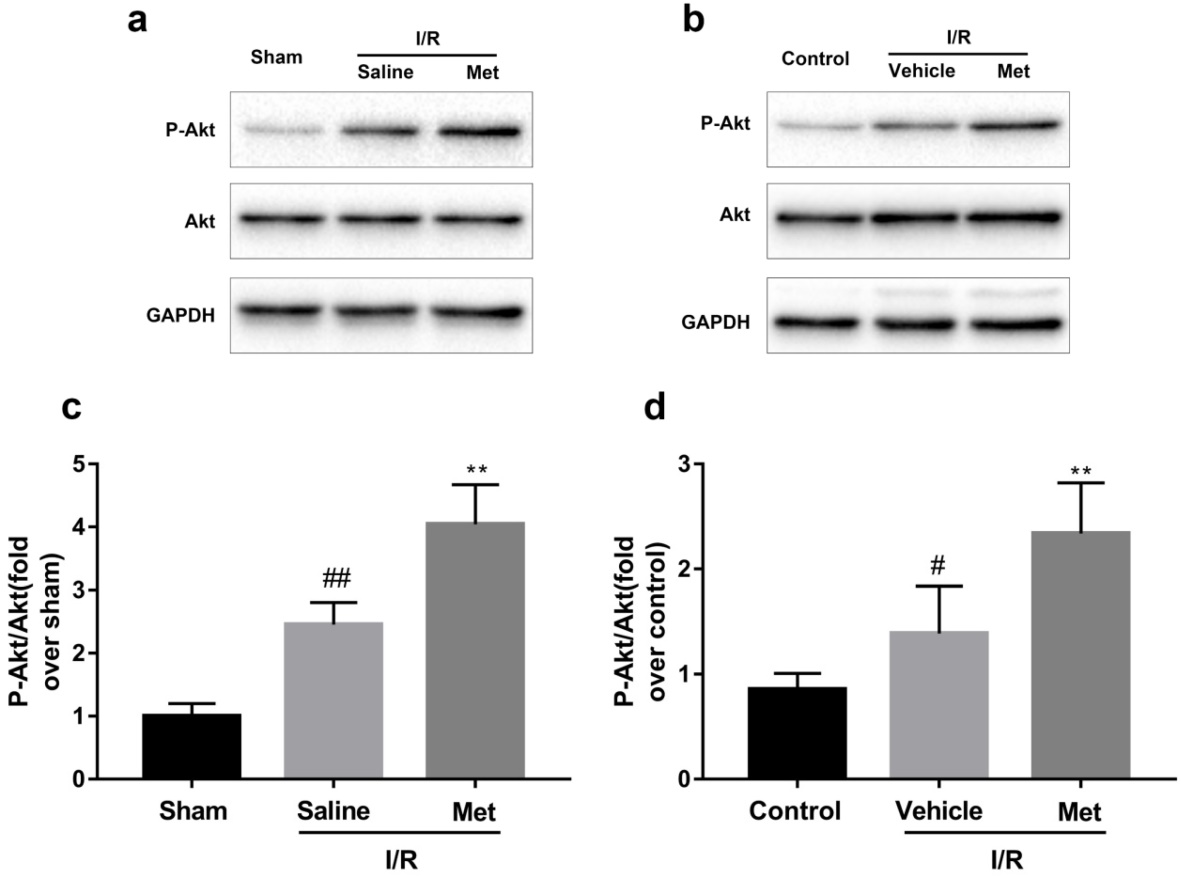
** Met increases phosphorylation of Akt during myocardial I/R.** Mice were treated with Met (125 µg/kg; i.v.) 15 min prior to ischemia. (**a, c**) Western blot analysis of p-Akt/Akt in mice myocardium after reperfusion for 4h. H9C2 cells were pretreated with Met (50 µM) for 12h then subjected to I/R injury for detection of (**b, d**) Western blot of p-Akt/Akt. n = 6. Values are expressed as the means ± SD. #p<0.05, ##p<0.01vs. the sham or control group, *p<0.05, **p<0.01vs. IR group, ^p<0.05, ^^p<0.01 vs. IR+Met group.

As shown in Figure 5a-d, compared with the control or sham group, the I/R group had markedly higher ratio of p-Akt to Akt, but not altered the total Akt, indicating that I/R injury activates the PI3K/Akt pathway both *in vivo* and *in vitro*. However, pretreatment with Met induced a higher ratio of p-Akt/Akt.

### Met-mediated autophagy depends on activation of Akt signaling pathway

To detect whether metformin inhibits autophagy during I/R injury via Akt signaling pathway, we employed miransertib or Akt-KO (CRISPR/Cas9) to inhibit Akt, both *in vivo* or in cultured primary cardiomyocytes. Our experiment showed that miransertib or Akt-KO could significantly reduce the level of Akt as well as reversed the effect of Met on the downregulation of LC3BII. Furthermore, the results were also demonstrated by immunofluorescence staining for LC3B (frozen cardiac sections) and MDC staining (cardiomyocytes). These findings suggested that Met-mediated autophagy depends on the activation of Akt signaling pathway.

## Discussion

Acute myocardial infarction (AMI) is one of the main causes of death threatening human life 29. Traditional reperfusion therapy inevitably leads to I/R injury, which will lead to rapid deterioration of heart function 30, 31. This study is the first to demonstrate that Met reduces apoptosis and inflammation induced by myocardial I/R injury through dampening autophagy in an Akt-dependent manner both *in vivo* and *in vitro*.

A growing number of studies have confirmed the activation of autophagy levels in many pathological states of the cardiovascular system, such as myocardial hypertrophy 32, heart failure 33, atherosclerosis 34 as well as myocardial I/R injury 35. Met, a classic drug for type 2 diabetes; has been shown to interfere with numerous human diseases independent of its antihyperglycemic effects and insulin-sensitizing effects 12, 36. Chronic Met treatment can alleviate myocardial apoptosis, fibrosis and improve cardiac function in diabetic mice by enhancing myocardial autophagy 9, 14. In addition, acute, low-dose Met therapy was able to improve myocardial I/R injury in nondiabetic and diabetic mice 20. However, to the best of our knowledge, there is no evidence that acute Met therapy can improve myocardial I/R injury through its regulating effects on autophagy.

Myocardial ischemia and reperfusion can trigger necrosis and apoptosis, both of which can lead to the loss of myocardial cells and thus reduce cardiac function 37. Cell necrosis releases large amounts of intracellular material into the surrounding tissue space, which activates inflammation and exacerbates the destruction of the surrounding cells 38. In recent years, emerging studies have confirmed that autophagy plays an important role in myocardial I/R injury 4. Autophagy is a vital physiological process in cells, which can degrade longevity proteins and dysfunctional organelles 39. Under certain conditions, autophagy induced by external stimuli promotes cell survival, but excessive and long-term autophagy is bound to lead to cell death 4. Autophagy plays a dual role between inflammation and apoptosis. First, autophagy can effectively remove inflammasome and inhibit the activity of inflammatory transcription factors, such as NF-κB 40. However, excessive autophagy may lead to the release of inflammatory factors 41. The nutrient status, such as starvation, induces phosphorylation of Bcl-2, resulting in dissociation of Bcl-2 from Beclin-1. Consequently, autophagy is activated and apoptosis is inhibited 42. On the other hand, autophagy related proteins Atg5 and Beclin-1 may turn into a pro-apoptotic protein in the case of being proteolyzed by proteases such as calpain 43, 44.However, whether autophagy plays a beneficial or harmful role in myocardial I/R injury has been a subject of great controversy. In the present study, we discovered autophagy performed a destructive role in I/R injury (Supplementary Figure 1a to 1g).

Both experimental and clinical studies confirm that Met holds the ability to protect the heart from impairment of hyperglycemia 45, 46. It seems that cardioprotective effect of Met is closely related to its hypoglycemic effect, however, in recent years, a great deal of studies have found that Met has additional potential mechanisms 36. For example, Met reduces myocardial infarct size by inhibiting mPTP opening through a PI3K-Akt-dependent manner 12. Meanwhile, a recent study demonstrated that a single, low-dose Met therapy activated AMPK-eNOS-mediated signaling and conferred cardioprotection against myocardial I/R injury 20. More recently, Xie and colleagues found that Met improved cardiac function by boosting myocardial autophagy in diabetic OVE26 mice 14.

In the present study, we found that Met attenuated I/R injury, and phenotypically, Met attenuated cardiomyocyte apoptosis, inflammatory responses during myocardial I/R injury which were due to Met-mediated alleviation of autophagy. Meanwhile, these beneficial effects could be reversed by inhibition of autophagosome formation (with rapamycin) or autophagosome processing(with CQ), illustrating that the cardioprotection of Met was mediated by reducing autophagosome formation and restoring autophagosome processing. Previous study demonstrated that the accelerated death of cells occurred mainly in the reperfusion stage rather than in the ischemic stage 47. Myocardial ischemia significantly increased AMPK phosphorylation, but it gradually returned to baseline after reperfusion 16, 17. However, the phosphorylation of Akt gradually elevated in both stage, and together with Beclin-1, Akt might play a crucial role in managing autophagy during reperfusion 16, 17. Similar to our research, our data showed that I/R markedly increased the phosphorylation of Akt but not an increase in AMPK phosphorylation (Supplementary Figurex1h to 1k). It may be due to the activation of Akt which can inhibit the activity of AMPK and this effect could be reverse in the presence of the AMPK activator, Met 48. It has also been reported that the activation of AMPK in the early stage of reperfusion promotes fatty acid oxidation and thus inhibits glucose utilization, leading to myocardial damage 49. In view of these findings, this study selected Akt to explore the in-depth mechanism of Met in attenuating myocardial I/R injury. To the best of our knowledge, it is the first to reveal the potential mechanism of Met in protecting the heart from I/R injury, especially through its dampening effects on autophagy in an Akt-dependent manner.

However, some of our conclusions seemed to conflict with other studies. First, great deals of studies have verified that Met has a protective effect by activating autophagy via the AMPK pathway in various diseases 9, 14. The opposite results might be possibly in part because the different disease models we employed. The pathological characteristics of different disease are doomed to be different, and autophagy as well as AMPK plays different roles in these processes. For example, autophagy is beneficial in spinal cord injury or diabetic cardiomyopathy, and phosphorylation of AMPK directly activates autophagy. But for myocardial I/R injury, at least in our model, autophagy is damaging and excessive autophagy might be the result of over-activation of AMPK 50. However, there was no obvious high expression of AMPK in I/R group in our study. What is more, previous studies have confirmed that AMPK mainly regulates autophagy during ischemia; however, Akt and Beclin-1 are responsible for domination of autophagy during reperfusion 16, 17. Second, Calvert and colleagues found that Met injected during reperfusion could conduct an activation of AMPK but not alteration of Akt 20. Bhamra demonstrated that Met administered pre-ischemically failed to activate Akt, however, Met administered at the time of myocardial reperfusion was able to enhance the expression of Akt but there was no significant difference on AMPK phosphorylation 12.In addition, another study discovered that Met added prior to the ischemia activated both Akt and AMPK signaling pathway 11. The mechanism for these different results remains unclear, but it seems that which myocardial I/R protocol (Langendorff perfusion or surgical ligation of the left coronary artery) we chose and the timing of the Met administration (prior to the ischemia or during reperfusion) play a key role.

The potential limitation of our study is that we only studied the protective effect of acute, low dose Met preconditioning on myocardial I/R injury. But in fact, whether Met injected during reperfusion has the same or other autophagy-related protective mechanisms also seems to be of great necessary. And studies had shown that chronic Met therapy was of cardioprotection 14, and chronic activation of Akt lost cardioprotective function 51, so we suspect that in our model, whether chronic Met treatment can lead to chronic Akt activation, and then whether it will affect the changes of autophagy and thus protect the myocardium? This will be an interesting topic. In addition, it would be appreciated to further clarify our views by replacing pharmacological inhibitors with transgenic mice.

In summary, our study confirms for the first time that pre-ischemic injection of an acute, single, low-dose Met attenuates myocardial autophagy, thereby inhibiting inflammation and apoptosis, and protects against myocardial I/R injury. This cardioprotective effect is related to activate Akt signaling pathway.

## Supplementary Material

Supplementary figures and tables.

## Figures and Tables

**Figure 6:**
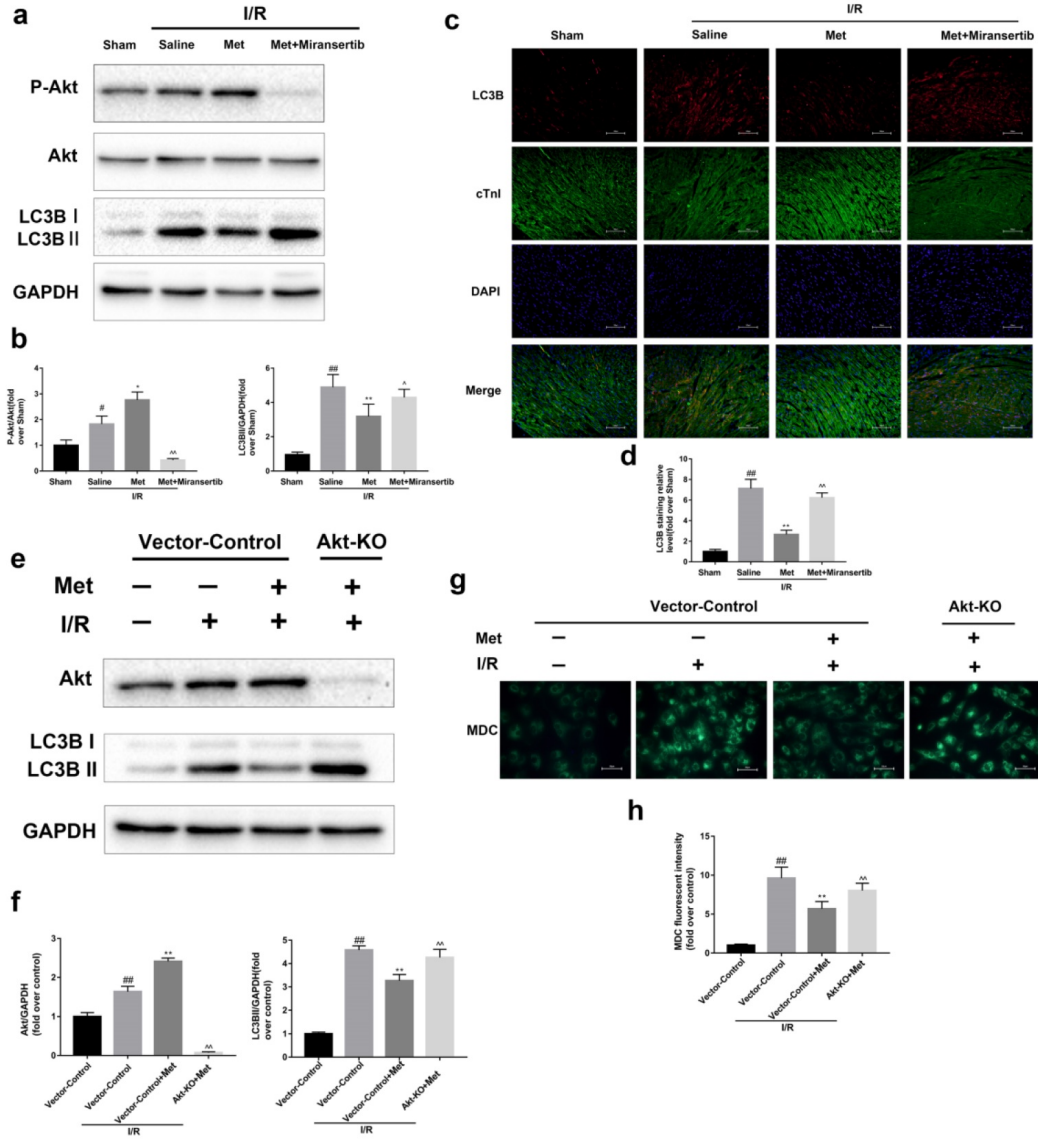
** Activation of Akt contributes to the regulation of I/R-induced autophagy by Met.** Mice were pretreated with Miransertib (120 mg/kg; i.p.) 1h before ischemia and Met (125 µg/kg; i.v.) 15 min prior to ischemia (**a, b**) Western blot analysis of p-Akt/Akt and LC3B. (**c, d**) Representative immunofluorescent images of staining with LC3-II (red), cTnI (green) and DAPI (blue) in the heart tissue. Scale bar: 100 µm.CRISPR/Cas9 system was used to knockout Akt in cultured primary cardiomyocytes and cells were pretreated with Met (50 µM) prior to I/R injury. (**e, f**) Western blot analysis of Akt and LC3B in cardiomyocytes. (**g, h**) Representative immunofluorescence images of cardiomyocytes loaded with MDC. Scale bar: 25 µm. n = 6. Values are expressed as the means ± SD. #p<0.05, ##p<0.01 vs. the sham or Vector-control group, *p<0.05, **p<0.01 vs. IR group, ^p<0.05, ^^p<0.01 vs. IR+Met group.
