# Supplementary material for: Metformin suppresses inflammation and apoptosis of myocardiocytes by inhibiting autophagy in a model of ischemia-reperfusion injury
Source: Int J Biol Sci. 2020 Jul 19;16(14):2559–79. doi: 10.7150/ijbs.40823 (PMC7415420; doi:10.7150/ijbs.40823)
Supplement: Supplementary file 1 — Supplementary figures and tables. [file ijbsv16p2559s1.pdf]

Supplementary data

Metformin suppresses inflammation and apoptosis of myocardiocytes by inhibiting autophagy in a model of ischemia-reperfusion injury

Running title: Metformin relieves myocardial ischemia/reperfusion via suppression of autophagy

Kai-yu Huang1 $, Jia-qun Que1$, Ze-song Hu2, Yong-wei Yu1, Ying-ying Zhou3, Lei Wang2, Yang-jing Xue1, Kang-ting Ji1# and Xin-min Zhang1#

1Department of Cardiology, The Second Affiliated Hospital and Yuying Children's Hospital of Wenzhou Medical University, Wenzhou 325027, Zhejiang, China

2The Second School of Medicine, Wenzhou Medical University, Wenzhou 325027, Zhejiang, China

3Department of Endocrinology, The Second Affiliated Hospital and Yuying Children's Hospital of Wenzhou Medical University, Wenzhou 325027, Zhejiang, China

$These two authors contributed equally to this work.

# Co-corresponding Authors:

Kang-ting Ji, MD, Department of Cardiology, The Second Affiliated Hospital and Yuying Children's Hospital of Wenzhou Medical University, Xueyuanxi Road, No 109, Wenzhou 325027, Zhejiang, China.Tel: 86-577-88002214; Fax: 86-577-88002214; E-mail:jikt@wzmc.edu.cn

Xin-min Zhang, MD, Department of Cardiology, The Second Affiliated Hospital and Yuying Children's Hospital of Wenzhou Medical University, Xueyuanxi Road, No 109, Wenzhou 325027, Zhejiang, China.Tel: 86-577-88002214; Fax: 86-577-88002214; E-mail: zhxinming@163.com

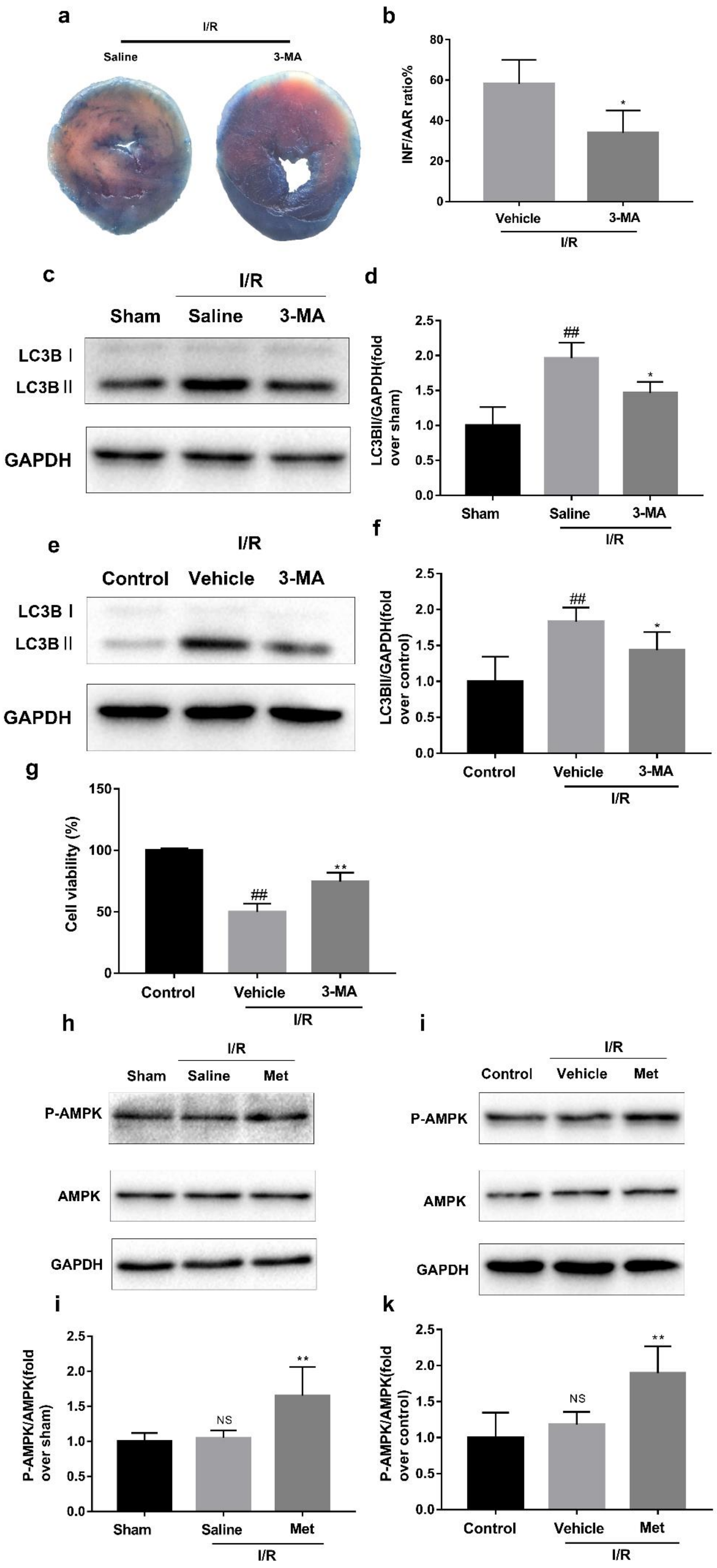

a
I/R
Saline
3-MA
b
INF/AAR ratio%
80
60
40
20
0
Vehicle
3-MA
I/R
c
I/R
Sham
Saline
3-MA
LC3B I
LC3B II
GAPDH
d
LC3BII/GAPDH(fold over sham)
##
Sham
Saline
3-MA
I/R
e
I/R
Control
Vehicle
3-MA
LC3B I
LC3B II
GAPDH
f
LC3BII/GAPDH(fold over control)
##
Control
Vehicle
3-MA
I/R
g
Cell viability (%)
150
100
50
0
##
**
Control
Vehicle
3-MA
I/R
h
I/R
Sham
Saline
Met
P-AMPK
AMPK
GAPDH
i
I/R
Control
Vehicle
Met
P-AMPK
AMPK
GAPDH
i
P-AMPK/AMPK(fold over sham)
NS
**
Sham
Saline
Met
I/R
k
P-AMPK/AMPK(fold over control)
NS
**
Control
Vehicle
Met
I/R

Supplementary figure legends

Supplementary Figure S1. Mice were pretreated with 3-MA (30mg·kg-1;i.p.)1h before ischemia and subjected to I/R injury, then killed at 4h after reperfusion for detection of (a, b) TTC stain, (c, d)Western blot indicating expression of LC3B protein. H9C2 cells were pretreated with 3-MA(10mM) for 2h then suffered from I/R injury for detection of (e, f)Western blot indicating expression of LC3B protein. (g)cell viability. Mice were treated with Met (125μg·kg-1;i.v.) 15 min prior to ischemia, (h, j) Western blot analysis of p-AMPK/AMPK in mice myocardium after reperfusion for 4h. H9C2 cells were pretreated with Met(50μM) for 12h then subjected to I/R injury for detection of(i, k) Western blot of p-AMPK/AMPK. n = 6. Values are expressed as the means ± SD. #$p<0.05$,##$p<0.01$vs. the sham or control group,*$p<0.05$,**$p<0.01$vs. IR group, ^$p<0.05$,^^$p<0.01$ vs. IR+Met group..
